# Metallo-supramolecular complexes enantioselectively target monkeypox virus RNA G-quadruplex and bolster immune responses against MPXV

**DOI:** 10.1093/nsr/nwae388

**Published:** 2024-10-30

**Authors:** Jie Yang, Geng Qin, Baoying Huang, Hualong Song, Jiewei Sun, Miles Postings, Peter Scott, Chuanqi Zhao, Chunyu Wang, Wenjie Tan, Jinsong Ren, Xiaogang Qu

**Affiliations:** Laboratory of Chemical Biology and State Key Laboratory of Rare Earth Resource Utilization, Changchun Institute of Applied Chemistry, Chinese Academy of Sciences, Changchun 130022, China; School of Applied Chemistry and Engineering, University of Science and Technology of China, Hefei 230026, China; Laboratory of Chemical Biology and State Key Laboratory of Rare Earth Resource Utilization, Changchun Institute of Applied Chemistry, Chinese Academy of Sciences, Changchun 130022, China; School of Applied Chemistry and Engineering, University of Science and Technology of China, Hefei 230026, China; NHC Key Laboratory of Biosafety, National Institute for Viral Disease Control and Prevention, Chinese Center for Disease Control and Prevention, National Key Laboratory of Intelligent Tracking and Forecasting for Infectious Diseases (NITFID), Beijing 102206, China; Department of Chemistry, University of Warwick, Coventry CV4 7AL, UK; NHC Key Laboratory of Biosafety, National Institute for Viral Disease Control and Prevention, Chinese Center for Disease Control and Prevention, National Key Laboratory of Intelligent Tracking and Forecasting for Infectious Diseases (NITFID), Beijing 102206, China; School of Pharmacy, Xinxiang Medical University, Xinxiang 453003, China; Department of Chemistry, University of Warwick, Coventry CV4 7AL, UK; Department of Chemistry, University of Warwick, Coventry CV4 7AL, UK; Laboratory of Chemical Biology and State Key Laboratory of Rare Earth Resource Utilization, Changchun Institute of Applied Chemistry, Chinese Academy of Sciences, Changchun 130022, China; School of Applied Chemistry and Engineering, University of Science and Technology of China, Hefei 230026, China; State Key Laboratory of Supramolecular Structure and Materials, Jilin University, Changchun 130012, China; NHC Key Laboratory of Biosafety, National Institute for Viral Disease Control and Prevention, Chinese Center for Disease Control and Prevention, National Key Laboratory of Intelligent Tracking and Forecasting for Infectious Diseases (NITFID), Beijing 102206, China; Laboratory of Chemical Biology and State Key Laboratory of Rare Earth Resource Utilization, Changchun Institute of Applied Chemistry, Chinese Academy of Sciences, Changchun 130022, China; School of Applied Chemistry and Engineering, University of Science and Technology of China, Hefei 230026, China; Laboratory of Chemical Biology and State Key Laboratory of Rare Earth Resource Utilization, Changchun Institute of Applied Chemistry, Chinese Academy of Sciences, Changchun 130022, China; School of Applied Chemistry and Engineering, University of Science and Technology of China, Hefei 230026, China

**Keywords:** G-quadruplex, ligand binding, Mpox virus, chirality, supramolecular chemistry

## Abstract

The Mpox virus (MPXV) has emerged as a formidable orthopoxvirus, posing an immense challenge to global public health. An understanding of the regulatory mechanisms of MPXV infection, replication and immune evasion will benefit the development of novel antiviral strategies. Despite the involvement of G-quadruplexes (G4s) in modulating the infection and replication processes of multiple viruses, their roles in the MPXV life cycle remain largely unknown. Here, we found a highly conservative and stable G4 in MPXV that acts as a positive regulatory element for viral immunodominant protein expression. Furthermore, by screening 42 optically pure chiral metal complexes, we identified the Λ enantiomer of a pair of chiral helical compounds that can selectively target mRNA G4 and enhance expression of the 39-kDa core protein encoded by the MPXV *A5L* gene. Mechanistically, RNA G4-specific helicase DHX36 inhibits A5L protein expression by unwinding G4s. In contrast, MH3 Λ enhanced mRNA stability by specifically targeting G4 structures and subsequently increased protein expression. Furthermore, given the pivotal role of the 39-kDa core protein in activating immune responses and facilitating virion maturation, modulation of MPXV G4 folding by MH3 Λ exhibited inhibitory effects on MPXV replication through enhancing the immune response. Our findings underscore the critical involvement of G4 in the MPXV life cycle and offer potential avenues for developing antiviral drugs that target G4.

## Teaser

This article identifies a highly conserved G-quadruplex (G4) in Mpox viruses, and found the chiral metallosupramolecular complex can enantioselectively target G4 and increase core protein expression, subsequently activating the host immune response.

## INTRODUCTION

The zoonotic disease Mpox (formerly monkeypox) is caused by infection with the Mpox virus (MPXV) and affects both humans and animals. MPXV is a kind of double-stranded DNA virus that belongs to the Poxviridae family orthopoxvirus (OPV) genus, which also includes variola virus (also known as smallpox), camelpox virus (CMPV) and vaccinia virus (VACV), etc. [1]. However, the disease has been known as a rare zoonotic infection for 50 years but limited investment and attention have been directed towards it beyond the African continent. Following the first reported infection in the UK on 13 May 2022, the outbreak expanded rapidly and now affects >100 non-endemic nations [1,2]. Although the World Health Organization declared that Mpox outbreaks no longer constituted a Public Health Emergency of International Concern in May 2023, it is worth noting that certain regions in Asia have witnessed an increase in Mpox cases due to the rapid evolution and increased international travel of the virus [3,4]. Although Mpox is not life-threatening, it is disruptive and painful for most patients, and there is currently no specific cure [5,6]. Smallpox vaccines have been trialed for the disease but supply is severely limited [6]. Several antivirals such as tecovirimat, brincidofovir, cidofovir and vaccinia immune globulin treatments that are based on smallpox and vaccinia are considered to be therapeutic options but not exclusively for MPXV [7,8]. Despite some advancements in the development of anti-Mpox drugs, there remains an imperative to expedite research on novel antiviral strategies that are characterized by heightened specificity and resistance inhibition.

G-quadruplexes (G4s) are atypical secondary structures of DNA or RNA that are composed of stacked layers of G-tetrad [9]. The structure is stabilized by π–π stacking, Hoogsteen hydrogen bonds and metal-ion coordination. There is increasing attention in the fields of chemistry and biology, as G4s play a pivotal role in regulating diverse biological processes [10,11]. In the past decade, G4s have been considered as therapeutic targets for multiple types of cancer [12], neurodegenerative diseases [13] and viruses [14–20], for example. The G4s in various viruses such as herpes simplex virus (HSV) [16], Epstein–Barr virus (EBV) [17], hepatitis B virus (HBV) [18], hepatitis C virus and SARS-CoV-2 [19,20], etc. have been shown to participate in regulation of the viral life cycle. Recently, our group reported the biological function of RNA G4s in the SARS-CoV-2 genome, whose formation can decrease viral protein expression and inhibit viral replication [19,20]. The G4 motif of the HBV virus is noteworthy for its ability to induce the expression of HBV proteins [18]. Interestingly, this is in contrast to the previous function of G4 in inhibiting protein expression and translation. Given these observations, it is urgent to explore whether G4s exist in the viral DNA or RNA of MPXV and their roles in the MPXV life cycle should be investigated.

Currently, >1000 G4 ligands have been characterized and several ligands have been tested for their antiviral properties [21]. While a number of small-molecule binding modes are known or have been postulated [22], typical synthetic G4 ligands contain flat aromatic chromophores for π–π stacking with G-quartets, perhaps with side chains for interaction with loops and grooves of the G4s, and steric bulk that prevents intercalation between base pairs [23]. Many of these compounds (such as TMPyP4, pyridostatin, telomestatin and BRACO-19) exhibit high affinity binding to G4s but their interaction is often mediated through an end-pasting mechanism and thus lacks specificity. The alternative G4 drug discovery strategy for the development of ligands that target rings and grooves is less selective for double-stranded DNA (dsDNA), which was the case for distamycin A and netropsin [24]. These contrast with natural proteins such as the helicase DHX36 that interacts with G4s principally via α-helical recognition units, with additional hydrogen-bonding to the adjacent DNA backbone [25]. We have developed several ranges of metallo-supramolecular assemblies whose diverse architectures are designed to emulate peptide α-helix units in terms of size, shape, charge and amphipathic nature, despite having very different underlying chemistries [26–32]. These metallo-supramolecular complexes are highly stable and resistant to unfolding and they are all readily available in both enantiomeric forms. This is a crucial factor in the efficacy and safety of drugs [33], as almost all biological targets nucleic acids and proteins are chiral and can be stereoselectively recognized by their binding ligands [34]. Correspondingly, we have found that metallo-supramolecular complexes selectively bind to telomeric G4s with a strong dependence on structure and stereochemistry [30,35–38]. Thus, we believe that there is great potential for such metallo-supramolecular complexes as selective G4 ligands.

In this study, by employing an integrated approach that encompasses bioinformatics, biophysical and biological techniques, we have successfully found a highly conserved RNA G4 structure within the MPXV *A5L* mRNA (encoding 39-kDa virion core protein) and elucidated its pivotal role as a positive regulatory element in viral immunodominant protein expression. Moreover, our findings underscore the impact on the A5L protein expression that results from modulation of the stability of this G4 through either chiral metallo-supramolecular complexes or RNA G4-specific helicase DHX36. More importantly, MH3 Λ exhibits selective binding to MPXV mRNA G4 rather than the numerous DNA G4s that are present in the human genome, thereby indicating the potential therapeutic applications of metallo-supramolecular complexes in Mpox therapy. Considering the pivotal role that is played by the *A5L* gene in immune response activation and virion maturation, targeted interaction of MH3 Λ with *A5L* mRNA G4 demonstrates remarkable antiviral effects through immune enhancement. Consequently, MPXV G4 has emerged as a promising target for innovative antiviral strategies, drugs and Mpox vaccines.

## RESULTS AND DISCUSSION

**Figure 1.**
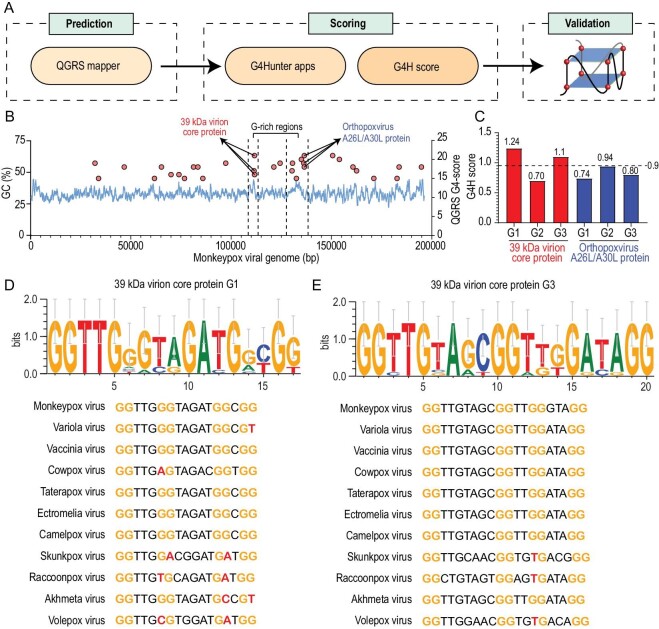
Bioinformatic and biophysical techniques used in G4s analysis. (A) Overall process of candidate discovery and validation. (B) GC% and QGRS G4 scores in preliminarily analysis of G4 regions. (C) G4H scores of potential G-quadruplex motifs to confirm G4 formation. (D) Conservation analysis of G1 sequence in orthopoxvirus. (E) Conservation analysis of G3 sequence in orthopoxvirus.

MPXV is a species of dsDNA virus whose ∼197-kb genome contains ∼190 non-overlapping open reading frames (ORFs) [39,40]. To investigate whether G4s exist in the MPXV genome, we first predicted the putative G4s sequences by using QGRS mapper software [41], evaluated the G4 folding capabilities by calculating the G4Hunter (G4H) scores [42] and finally confirmed the formation of G4s *in vitro* and in live cells via biophysical techniques (Fig. 1A). We found that the DNA G4s that are present have only two G-tetrad layers, which may be difficult to stabilize under physiological conditions. We thus focused on the more stable RNA G4s. A total of 29 putative viral mRNA G4 sequences were identified (Table S1). We found that only two of these (encoding 39-kDa virion core protein and A26L/A30L protein) are in the relatively G-rich region in the MPXV genome and contain three putative RNA G4 sequences (Fig. 1B). Furthermore, we evaluated the G4H scores of the six putative RNA G4 sequences and found that only the G4H scores of G1 and G3 were above the threshold (Fig. 1C). Finally, we assessed the levels of sequence conservation of these two G4s and found that G3 is highly conserved in the orthopoxvirus genome and well maintained through the MPXV strains (Fig. 1D and E, and Fig. S1A and B), suggesting a key role for G3 in the viral life cycle of MPXV. Thus, we chose G3 for an in-depth study.

**Figure 2.**
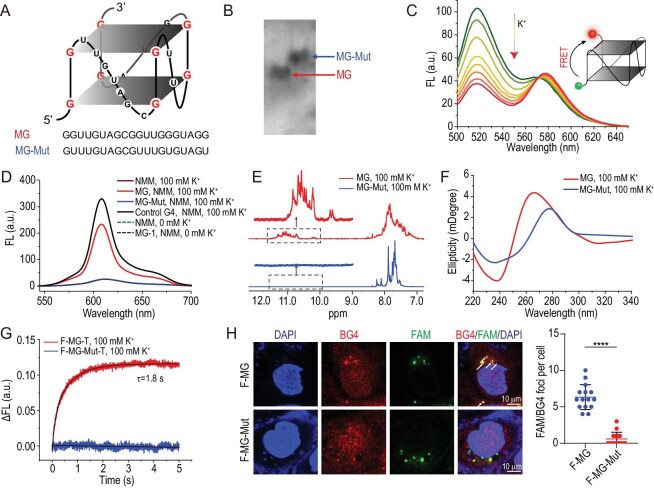
Confirmation of MG G4 formation and kinetic analysis. (A) Schematic diagram of the potential MG G4 structure. (B) Analysis of MG by natural gel electrophoresis. Lane 1, MG; Lane 2, MG-Mut. The concentration of all the RNAs is 2 μM. Samples were prepared in Tris-HCl buffer containing 100 mM KCl. (C) FRET between FAM and TAMRA labeled F-MG-T at various [K^+^] and excitation wavelengths was 492 nm. (D) Fluorescence turn-on assays of NMM under various conditions; NMM is 0.6 μM, RNA is 0.3 μM and excitation wavelength was 399 nm. (E) ^1^H NMR spectroscopy of MG G4 formation evidenced; resonances in the range of 6.8–12 ppm due to Hoogsteen imino peaks of MG G4. (F) CD spectra of MG and MG-Mut. (G) Stopped-flow traces of F-MG-T or F-MG-Mut-T samples when mixed with Tris-HCl buffer containing 200 mM [K^+^]; excitation wavelength 492 nm, data recorded at 578 nm. (H) Immunofluorescence assays of F-MG G4 in live cells. Left: the arrows indicate the co-localized foci of FAM-labeled RNA with BG4. Right: FAM/BG4 foci number was quantified. ****P < 0.0001.

To confirm whether the selected G3 could form a stable G4 structure *in vitro* and in live cells, we synthesized MPXV G3 RNA (MG), 5′-FAM-modified MPXV G3 RNA (F-MG), 5′-FAM, 3′-TAMRA-modified MPXV G3 RNA (F-MG-T) and their corresponding mutants (MG-Mut, F-MG-Mut and F-MG-Mut-T) in advance (Fig. 2A and Table S2). Firstly, native polyacrylamide gel electrophoresis (PAGE) assays and fluorescence resonance energy transfer analysis (FRET) [43] can be used to detect the secondary structure formation of nucleic acid chains. The results of PAGE assays showed that MG migrated faster than MG-Mut (Fig. 2B) and the FRET assays of F-MG-T indicated the FRET between FAM (donor) and TAMRA (acceptor) under an enhanced K^+^ concentration, suggesting that MG forms a secondary structure that is different from the single strand in the presence of K^+^ (Fig. 2C). In addition, we employed *N*-methyl mesoporphyrin IX (NMM) fluorescence on the assay for preliminary verification of the G4 formation. The results showed that the formation of RNA G4 in the presence of K^+^ was confirmed by an obvious enhancement in the NMM fluorescence intensity and neuroblastoma RAS viral oncogene homolog (NRAS) RNA as a positive control showed the same result whereas the mutant did not (Fig. 2D). Meanwhile, ^1^H nuclear magnetic resonance (NMR) results showed that MG had obvious peaks in the chemical-shifts region of G4s (10.2–12.5 ppm) (Fig. 2E), further supporting the proposal that MG can fold into a G4 structure. Circular dichroism (CD) spectra of MG indicated the formation of a parallel structure G4 with a positive peak at 263 nm and a negative peak at 240 nm, while no characteristic G4 peaks appeared in the CD spectra of MG-Mut (Fig. 2F). In addition, the lack of change in the measured melting temperature (*T*_m_) with RNA concentration (Fig. S2) indicates that the G4 structure is a result of intramolecular folding.

We next investigated the kinetic and thermodynamic processes that are involved in the formation of MG G4. Stopped-flow assays showed that the fluorescence intensity reached a plateau at ∼1.8 s, indicating that MG G4 formation was essentially complete within that time, i.e. moderate folding kinetics (Fig. 2G). Meanwhile, thermal melting methods (Fig. S3) indicated that the G4 formation of MG was driven by enthalpy–entropy compensation, while the −4.39 kcal mol^−1^ Δ*G*°_25_ was mainly due to its two G-tetrads unit structure.

The formation of MG G4 in buffer encouraged us to further investigate the formation of MG G4 in live cells. To this end, we transfected F-MG and F-MG-Mut RNAs into MCF-7 cells and G4 formation was monitored via immunofluorescence assays by using a specific G4-binding antibody, BG4. We stained the cell nucleus with 4′,6-diamidino-2-phenylindole (DAPI) (#JL-R3329, Jianglai biology, Shanghai) and observed the distribution of G4 in the cells through red fluorescence. Indeed, we observed significant colocalization between F-MG and BG4 but not between F-MG-Mut and BG4 (Fig. 2H), indicating the ability of MG G4 to form G4 structures in living cells. Although it may differ from the status of the MG sequence in MPXV, the presence of a short sequence in a G4 structure within living cells provides promising evidence for further investigation into its role in the viral life cycle. Furthermore, we employed BG4 antibody to assess the alterations in the G4 content during MPXV infection. Our results indicate that G4 levels in cells were significantly upregulated with increasing virus multiplicity of infection, suggesting that G4 plays a critical role in the viral infection cycle (Fig. S4).

**Figure 3.**
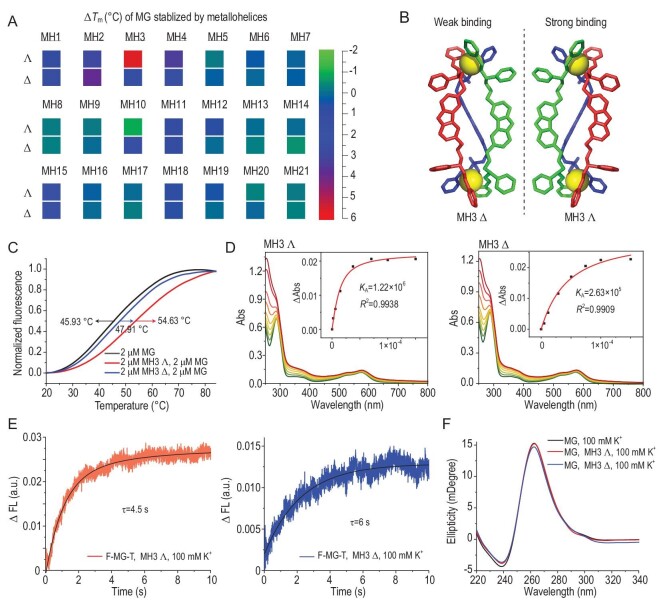
Stabilization of MG by metallo-supramolecular complexes MH3. (A) Melting temperature change (Δ*T*_m_) of MG stabilized by metallo-supramolecular complexes. (B) Structures of metallo-supramolecular complexes MH3. (C) Fluorescence thermal melting curves of F-MG-T (2 μM) in the absence of MH3 Λ or MH3 Δ (2 μM). (D) Absorption spectra of MH3 Λ (left) and MH3 Δ (right) at increasing concentrations of MG. Inset: plot of ΔAbs at 574 nm versus concentration of MG. (E) Typical stopped-flow traces of F-MG-T mixed with MH3 Λ (left) or MH3 Δ (right). (F) CD spectra of the MH3 Λ and MH3 Δ in the absence of MG in 100 mM KCl.

Traditional small-molecule G4 ligands generally show poor drug-like properties and selectivity profiles, which limits their application. We have demonstrated the potential for selective targeting of G4s by using our chiral metallo-supramolecular complexes [30,34–38]. The wide range of architectures, shapes, sizes and functionalities that are available [26–31] and our ability to access both enantiomers of each structure provide a unique opportunity to discover a specific peptide-mimetic [32] binder and stabilize MG G4. We thus screened a diverse selection from our library (Fig. 3A and Figs S5–S25). Among 42 optically pure complexes, we identified a pair of enantiomers (the Λ-enantiomer MH3 Λ and Δ-enantiomer MH3 Δ) that bind and stabilize MG G4 with enantioselectivity (Fig. 3B), which is a clear indicator that rather subtle aspects of the structure, such as the disposition of charge and π-stacking motifs, are involved in binding. Melting profiles of the 5′-FAM and 3′-TAMRA-labeled F-MG-T probe were detected under varying conditions. With fluorescence as a function of temperature, G4 thermal stability was measured by using a real-time polymerase chain reaction (rt-PCR) machine under varying control and test conditions. Relative fluorescence units were normalized to initial (20°C) and final (85°C) values [43]. We found that both MH3 enantiomers stabilize MG G4 but MH3 Λ was the stronger, and the difference Δ*T*_m_ between the MH3 Λ and MH3 Δ enantiomers reached 6.7°C (Fig. 3C). Further, the thermodynamic parameters of the binding between the metallohelix enantiomers and the MG G4 were estimated by using thermal melting methods (Fig. S26), indicating a contribution from entropic and enthalpic factors. Consistently with this, the binding constants with MG that were determined by using ultraviolet (UV)-visible spectroscopy titration were estimated to be 1.22 × 10^6^ M^−1^ for MH3 Λ and approximately one magnitude lower at 2.63 × 10^5^ M^−1^ for MH3 Δ (Fig. 3D). The detailed UV-titration analysis indicated a 1:1 binding model for the ligand/MG complex. Reaction times *τ* that were calculated from stopped-flow measurements also showed that MH3 Λ binding was faster (Fig. 3E). In addition, the CD characteristic peak position of MG G4 was unchanged in the presence of MH3 Λ or MH3 Δ (Fig. 3F), implying that enantiomer binding did not destroy the parallel structure of the G-quadruplex. By considering MH3 Λ or other metallohelices as therapeutic entities in this context, we need to determine their preferential binding to G4 RNA of MPXV rather than the abundant DNA G4 that is present in the cell. For this purpose, a number of G4 DNAs were used for FRET-melting experiments. The results showed no significant binding of MH3 Λ or MH3 Δ to DNA G4 (Fig. S27 and Table S3), which may greatly reduce off-target risk for therapeutic use. Taken together, these results indicate that the chiral metallo-supramolecular complexes MH3 Λ and MH3 Δ enantioselectively target MG G4, with the Λ-metallo-supramolecular complex displaying the stronger binding capacity and better effects of stabilization. In addition, when compared with a variety of common G4 ligands (Fig. S28), the markedly enhanced stability of MG upon MH3 Λ binding further underscores the selectivity of MH3 Λ for MG.

**Figure 4.**
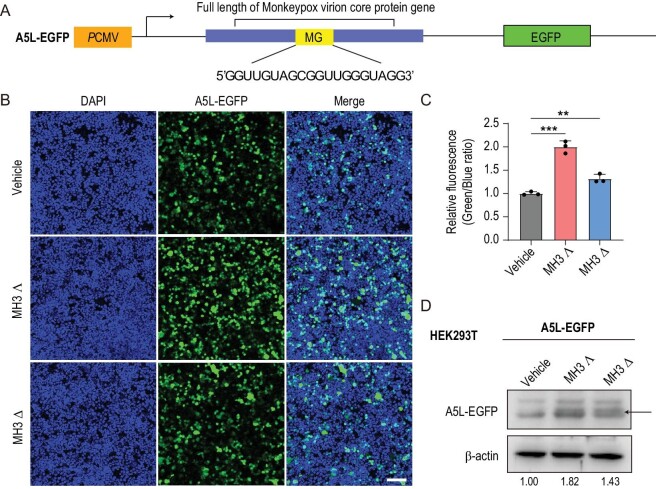
Modulating the formation of MG G4 by metallo-supramolecular complexes increases the protein level of the core protein by promoting its translation in cells. (A) Schematic diagram of the constructed A5L–EGFP reporter gene plasmids. (B–D) Effects of MH3 Λ or MH3 Δ on the expression of A5L–EGFP reporter (B: confocal pictures, C: relative fluorescence intensity analysed by using ImageJ, D: Western blotting). The relative grayscale values of A5L–EGFP protein were calculated by using ImageJ and labeled below the corresponding bands. **P < 0.01; ***P < 0.001. Bars = 100 μm.

These findings prompted us to investigate the roles of MG G4 in the viral life cycle of MPXV by using both MH3 Λ and MH3 Δ. Initially, we assessed the impact of MH3 Λ and MH3 Δ on cell viability to determine an appropriate administration concentration by CCK-8 assay (#CT0001, Shandong Sparkjade Biotechnology Co., Ltd) (Figs S29 and S30). Subsequently, considering the localization of MG G4 within the mRNA sequence of the *A5L* gene, we postulate that the presence of MG G4 might play a role in modulating the expression of the protein encoded by *A5L*. To address this, we cloned the sequences of MG and MG-Mut into a pLV–EGFP-N vector and transfected it as a reporter into HEK293T cells (Fig. S31). Subsequently, we harvested the cells from the 24-well plate (NEST Biotechnology, China) and observed an increase in the protein expression of MG-WT–EGFP following treatment with MH3 Λ or MH3 Δ, while no such effect was observed for MG-Mut–EGFP (Figs S32–S34), indicating that these enantiomers enhance protein expression by targeting the G4 structure. Consistently with these findings, MH3 Λ exhibited higher levels of protein induction compared with MH3 Δ (Fig. S33). Subsequently, we successfully cloned the complete *A5L* sequence into the pLV–EGFP-N vector and introduced the reporter gene into HEK293T cells (Fig. 4A). Importantly, no other G-quadruplex-forming motifs were identified within the enhancer and promoter regions of *A5L–EGFP* mRNA or in the reporter plasmid (Table S4). When we treated cells with MH3 Λ or MH3 Δ at different concentrations, both confocal and Western blot assays demonstrated that the compounds can upregulate the expression of A5L–EGFP fusion proteins, suggesting that the MG G4 formation promotes the protein expression of *A5L* (Fig. 4B–D and Figs S35 and S36). It is noteworthy that the difference in binding ability between the enantiomers and G4 also led to the different expression of A5L–EGFP fusion proteins, further indicating the MH3 Λ enantioselectivity (Fig. 4B–D). In addition, to further analyse the role of G4 in A5L expression, we constructed the A5L-Mut–EGFP fluorescent reporter vector. In this context, we introduced a mutation into the MG sequence of the A5L–EGFP fluorescent reporter vector that did not affect the expression of the 39-kDa protein (Fig. S37A). We first demonstrated that MG-Mut2 could not form G4 structures *in vitro* by using CD spectroscopy and gel electrophoresis (Fig. S37B and C). Furthermore, the addition of MH3 compounds did not alter the expression of A5L–EGFP protein in cells that were transfected with the A5L-Mut–EGFP fluorescent reporter vector (Fig. S37D) under our experimental conditions. Our results further illustrated the role of the MG G4 structure in the protein expression.

**Figure 5.**
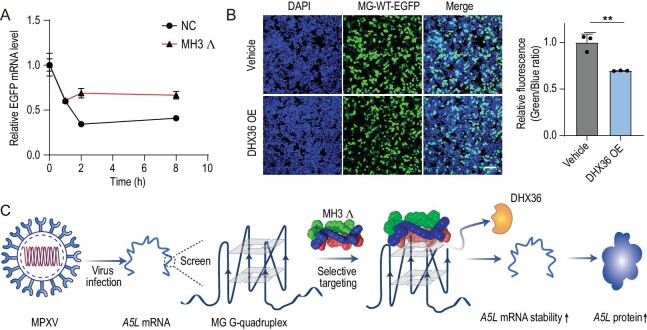
MH3 Λ enhances RNA stability by displacing DHX36, a G4-interacting protein. (A) The addition of MH3 Λ can significantly enhance the stability of MG–EGFP RNA. (B) Effect of overexpression of DHX36 on EGFP expression in cells transfected with MG-WT–EGFP reporter by confocal laser scanning microscope (B left: confocal pictures; B right: relative fluorescence intensity analysed by using ImageJ). **P < 0.01. Bars = 100 μm. (C) Schematic representation of the MH3 Λ-mediated upregulation of A5L protein via its interaction with G4.

In order to further elucidate the mechanism by which MG G4 regulates A5L protein, we selected the ligand MH3 Λ with enhanced binding affinity to MG for subsequent investigation. It was observed that the addition of MH3 Λ significantly augmented the stability of *EGFP* mRNA in cells that were transfected with MG-WT–EGFP (Fig. 5A). Additionally, we employed DHX36, which is an RNA G4-specific helicase, to unwind MG G4 structures. Our findings demonstrated that overexpression of DHX36 inhibited MG-WT–EGFP protein expression (Fig. 5B) but had no effect on MG-Mut–EGFP (Fig. S38). In addition, we employed siRNA to knock down the DHX36 protein within the transfected fluorescent reporter system, which was validated by using Western blotting (Fig. S39A and D). The knock-down of DHX36 resulted in an upregulation of MG-WT–EGFP protein expression in cells that were transfected with the MG-WT–EGFP fluorescent reporter vector, while having no observable effect on MG-Mut–EGFP (Fig. S39B, C, E and F). These results suggest that DHX36 helicase may participate in the regulation of the *A5L* gene as an unwinding protein for G4 structures, thereby downregulating A5L protein levels. Collectively, these findings indicate that the MH 3Λ metal spiral might compete with host RNA helicases DHX36 to stabilize MPXV G4 structures and enhance *A5L* mRNA stability, thereby augmenting 39-kDa core protein expression (Fig. 5C).

**Figure 6.**
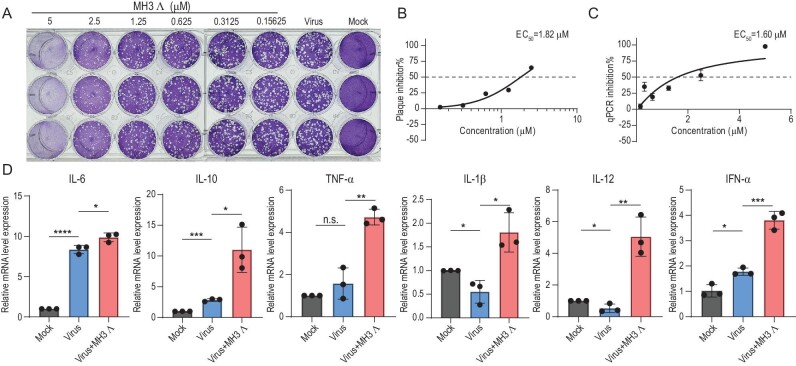
The antiviral efficacy of MH3 Λ against MPXV. (A) Representative plaques were mapped after treatment with varying concentrations of MH3 Λ. (B) Quantitative representation of plaque-forming units is depicted. The percentage of plaque formation in the presence of varying concentrations of MH3 Λ inhibitor is expressed relative to the number of plaques observed without the inhibitor. (C) The antiviral efficacy of MH3 Λ was assessed via qPCR. (D) Effect of MH3 Λ on the mRNA expression levels of IL-6, IL-10, TNF-α, IL-1β, IL-12 and IFN-α in Vero cells infected with MPXV. Data shown are mean ± SE; *n* = 3. *P < 0.05; **P < 0.01; ***P < 0.001; ****P < 0.0001. n.s., not significant.

The virion core protein, which is a 39-kDa product that is encoded by the *A5L* gene, is an immunogenic protein that is highly conserved in poxviruses. It plays a crucial role in activating the host immune response and facilitating the transition from intracellular virion (IV) to intracellular mature virion (IMV) [44,45]. We found that the G4 structure within the encoding mRNA of the *A5L* gene is involved in regulating the self-synthesis of 39-kDa virion core protein. MG G4 formation promoted the expression of the 39-kDa virion core protein, while MG G4 unfolding inhibits the protein expression, suggesting that MG G4 acts as critical positive regulatory element of viral immunodominant protein. Given the pivotal role of the core protein in the life cycle of MPXV, our objective is to investigate whether MH3 Λ, which is a metal helical compound with enhanced regulatory capability on proteins, can exert antiviral effects by specifically targeting the internal G4 binding site of the virus. Firstly, we assessed the cytotoxicity of MH3 Λ in Vero cells by performing CCK8 assays and determined that the half-cytotoxic concentration (CC_50_) of MH3 Λ was 10.27 μM (Fig. S40). Subsequently, considering the high conservation of MG across various orthopoxvirus, we preliminarily validated the antiviral effect of MH3 Λ against VACV. The safe dose of MH3 Λ was found to inhibit the green fluorescence intensity of VACV in Vero cells that were infected with TTV–EGFP, indicating its potential as an anti-VACV agent (Fig. S41). Furthermore, the antiviral effect of MH3 Λ against VACV was confirmed through qPCR and plaque-counting assays (Figs S42 and S43A). The inhibitory activity of MH3 Λ on TTV-Luv luciferase further supports its antiviral efficacy (Fig. S43B). Finally, we conducted MH3 Λ antiviral experiments by utilizing MPXV. Upon the addition of MH3 Λ at a safe concentration in Vero cells that were infected with MPXV, we observed a dose-dependent reduction in the viral infection activity (PFU) of MPXV (Fig. 6A and B) and the F3L-specific viral nucleic acid copy number (Fig. 6C), indicating the potential antiviral effect of MH3 Λ against MPXV. This antiviral activity can also be attributed to the immunogenicity that is induced by the core protein, which elicits a robust immune response (Fig. 6D), and the addition of MH3 alone did not affect the production of cellular immune factors under our experimental conditions (Fig. S44). These findings suggest that the stabilization of G4 by MH3 Λ may further augment *A5L* mRNA translation and bolster immune responses against MPXV.

Although this study demonstrated an antiviral effect of MH3 Λ, there are inherent limitations that need to be considered. Currently, some G4 ligands have been tested for their antiviral properties. However, due to the existence of G4s in both cell and virus genomes, the development of antiviral G4 ligands that specifically target viral G4s but not host cellular G4s has been a serious challenge [46,47]. It is still the major limitation of the G4 ligands that have been described so far. Only a few G4 ligands have shown selectivity between DNA and RNA G4 [48–50]. The selectivity of these metallohelices in targeting RNA G4 may reduce the off-target effects to a certain extent. Meanwhile, more and more studies have proposed that, during virus infection in the host, the virus will copy and transcribe its genome in large quantities, producing a large amount of viral RNA G4s that is far beyond that of the cell host RNA G4s, which may compensate for the limited specificity of the ligands in host RNA G4s [14,46]. For example, during the replication of herpes simplex virus 1(HSV-1) [16] and SARS-CoV-2 [20], the number of virus G4s increases dramatically so the off-target effects might be reduced in large part. Our study suggests that modulation of the unfolding or folding of MPXV RNA G4 could potentially enhance the immune response by influencing RNA stability and preventing immune evasion, thereby achieving antiviral effects against MPXV. Importantly, the identification of novel targets that are based on G4 structures in viral mRNA holds promise for the development of G4-based anti-MPXV drugs.

## CONCLUSIONS

In summary, the development of novel antiviral strategies, drugs and vaccines against MPXV is urgently needed. In this work, we identified a highly conserved and stable G4 motif in MPXV and demonstrated its role as a positive regulatory element for the expression of viral immunodominant proteins, highlighting its potential as an antiviral target. Furthermore, we demonstrated that destabilization of the G4 structure by using the G4-specific helicase DHX36 can effectively inhibit the protein expression of the MPXV *A5L* gene. Conversely, the Λ-enantiomer MH3 Λ in chiral metallo-supramolecular complexes competes with helicase DHX36 to stabilize G4s, enhance mRNA stability and increase A5L protein expression, thereby exhibiting potent antiviral effects against MPXV. These findings underscore the significance of G4s in viral mRNA as crucial regulatory signals for viral immunodominant protein expression and immune evasion. The insights that have been gained from this research provide novel perspectives on the biological function of G4s in the lifecycle of MPXV and offer potential avenues for the development of antiviral therapeutics that target Mpox prevention and treatment.

## Supplementary Material

nwae388_Supplemental_File
