# Supplementary material for: Metallo-supramolecular complexes enantioselectively target monkeypox virus RNA G-quadruplex and bolster immune responses against MPXV
Source: Natl Sci Rev. 2024 Oct 30;12(1):nwae388. doi: 10.1093/nsr/nwae388 (PMC11697978; doi:10.1093/nsr/nwae388)
Supplement: nwae388_Supplemental_File [file nwae388_supplemental_file.pdf]

Supporting Information for

Metallo-supramolecular Complexes Enantioselectively Target Monkeypox Virus RNA G-quadruplex and Bolster Immune Responses against MPXV

AUTHORS

Jie Yang,[1,2,+] Geng Qin,[*,1,2,+] Baoying Huang,[3,+] Hualong Song,[4] Jiewei Sun,[3,5] Miles Postings,[4] Peter Scott,[4] Chuanqi Zhao,[*,1,2] Chunyu Wang,[6] Wenjie Tan,[*,3] Jinsong Ren,[1,2] and Xiaogang Qu[*,1,2]

[1] Laboratory of Chemical Biology and State Key Laboratory of Rare Earth Resource Utilization, Changchun Institute of Applied Chemistry, Chinese Academy of Sciences, Changchun, Jilin 130022, P. R. China.

[2] University of Science and Technology of China, Hefei, Anhui 230026, P. R. China.

[3]NHC Key Laboratory of Biosafety, National Institute for Viral Disease Control and Prevention, Chinese Center for Disease Control and Prevention, Beijing, 102206, China； National Key Laboratory of Intelligent Tracking and Forecasting for Infectious Diseases (NITFID)

[4]Department of Chemistry, University of Warwick, Coventry CV4 7AL, UK.

[5]School of Pharmacy, Xinxiang Medical University, Xinxiang 453003, China.

[6]State Key Laboratory of Supramolecular Structure and Materials, Jilin University, Changchun, Jilin 130012, P. R. China.

[+]These authors contributed equally to this work.

*Correspondence: xqu@ciac.ac.cn

This PDF file includes:

Supporting text
Figures S1 to S44
Tables S1 to S4
SI References

Supporting Information Text

Materials and Methods 4

Figure S1 The conservation of the two G4 candidates 8

Figure S2 Fluorescence thermal melting curves of MG 9

Figure S3 Thermodynamic parameters of MG G4 formation 10

Figure S4. Immunofluorescence assays of MPXV infection in Vero cells 11

Figure S5 Effect of MH1 on thermal stabilization of MG G4 12

Figure S6 Effect of MH2 on thermal stabilization of MG G4 13

Figure S7 Effect of MH3 on thermal stabilization of MG G4 14

Figure S8 Effect of MH4 on thermal stabilization of MG G4 15

Figure S9 Effect of MH5 on thermal stabilization of MG G4 16

Figure S10 Effect of MH6 on thermal stabilization of MG G4 17

Figure S11 Effect of MH7 on thermal stabilization of MG G4 18

Figure S12 Effect of MH8 on thermal stabilization of MG G4 19

Figure S13 Effect of MH9 on thermal stabilization of MG G4 20

Figure S14 Effect of MH10 on thermal stabilization of MG G4 21

Figure S15 Effect of MH11 on thermal stabilization of MG G4 22

Figure S16 Effect of MH12 on thermal stabilization of MG G4 23

Figure S17 Effect of MH13 on thermal stabilization of MG G4 24

Figure S18 Effect of MH14 on thermal stabilization of MG G4 25

Figure S19 Effect of MH15 on thermal stabilization of MG G4 26

Figure S20 Effect of MH16 on thermal stabilization of MG G4 27

Figure S21 Effect of MH17 on thermal stabilization of MG G4 28

Figure S22 Effect of MH18 on thermal stabilization of MG G4 29

Figure S23 Effect of MH19 on thermal stabilization of MG G4 30

Figure S24 Effect of MH20 on thermal stabilization of MG G4 31

Figure S25 Effect of MH21 on thermal stabilization of MG G4 32

Figure S26 Thermodynamic parameters between chiral compounds and MG. 33

Figure S27 Effect of MH3 Λ and MH3 Δ on a range of different DNA G-quadruplexes 34

Figure S28 Effect of G4 ligands on thermal stabilization of MG G4 35

Figure S29 Toxicity analysis of MH3 Λ or MH3 Δ in MCF-7 36

Figure S30 Toxicity analysis of MH3 Λ or MH3 Δ in HEK293T 37

Figure S31 The schematic diagram of the constructed EGFP reporter gene plasmids 38

Figure S32 Effects of MH3 on the expression of EGFP reporter by western blot assays 39

Figure S33 Effects of MH3 on the expression of MG-WT-EGFP reporter 40

Figure S34 Effects of MH3 on the expression of MG-Mut-EGFP reporter 41

Figure S35 Effects of MH3 Λ on the expression of A5L-EGFP reporter 42

Figure S36 Effects of MH3 Δ on the expression of A5L-EGFP reporter 43

Figure S37 Effects of MH on the expression of A5L-Mut-EGFP reporter 44

Figure S38 Effect of overexpressed DHX36 on MG-Mut-EGFP reporter protein 45

Figure S39 Effect of DHX36 knockdown on EGFP reporter 46

Figure S40 Inhibition activity curve of MH3 Λ against vero cells 47

Figure S41 Effect of MH3Λ on the fluorescence intensity of vaccinia virus TTV-EGFP 48
Figure S42 Effect of MH3Λ on vaccinia virus TTV-EGFP 49
Figure S43 The antiviral activity of MH3Λ against vaccinia virus 50
Figure S44 Effect of MH3Λ on the expression levels of cytokines 51
Table S1 Characteristics of the 29 putative RNA G-quadruplexes 52
Table S2 Name and sequence of the oligonucleotides and primers used in this study 54
Table S3 Effect of addition of ligands on DNA G4 stability 56
Table S4 The sequence of A5L-EGFP mRNA and the plasmid enhancer-promoter 57
SI References 59

Materials and Methods

Methods

The Agents and Cell culture

The RNAs were obtained from Sangon (Shanghai, China). The chiral metal-supramolecular complexes were synthesized and characterized following previously reported methods(1-3). The chemicals were procured from Sigma–Aldrich. And all water employed for the preparation of buffer solutions was subjected to DEPC treatment.

Recombinant vaccinia virus expressing green fluorescent protein (TTV-EGFP) and MPXV (MPXV-B.1-China-C-tan-CQ01) were isolated and stored at Chinese Center for Disease Control and Prevention (China CDC). MCF-7 and HEK293T cells were cultured in DMEM (Gibco, UK) containing 10% FBS (Gibco), and Vero cells were cultured in MEM cell culture medium containing 10% FBS. The media were all supplemented with a 1% of penicillin and streptomycin. All the cells were cultured in a humidified 37 °C incubator with 5% CO_2.

Bioinformatics analysis

The full genome sequences of MPXV were acquired from the NCBI Genome database (https://www.ncbi.nlm.nih.gov/genomes). The subsequent step involved the utilization of GQRS-mapper and G4Hunter to predict putative G4-forming sequence. The search algorithm was based on the criteria of $G{\geq}2N_{1\text{-}15}G{\geq}2N_{1\text{-}15}G{\geq}2N_{1\text{-}15}G{\geq}2$, where 'G' represents guanine and 'N' represents any base including guanine."

Fluorescence assays

The assays were conducted at 25 °C using a JASCO FP-6500 spectrofluorometer. The NMM fluorescence turn-on assays were performed by incubating samples (400 μL) containing 1 mM NMM, 0.5 μM RNA strand, and 100 mM K^+ metal ions at 4 °C for 3 hours. The emission spectra of the mixture were then tested at an excitation wavelength of 399 nm and a temperature of 25 °C. The fluorescence resonance energy transfer analysis involved gradually adding a high concentration K^+ buffer to the F-MG-T solution. Spectra were recorded between 500 and 650 nm, with excitation at 492 nm.

Native-PAGE

The native-PAGE was performed on a 20% acrylamide gel, where RNAs labeled at the 5'-end with FAM were subjected to electrophoresis in 100 mM K^+ followed by gel electrophoresis at 4°C.

Circular dichroism (CD) spectroscopy measurements

The assays were conducted at 25 °C using a JASCO J-810 spectropolarimeter. The spectra of the complexes /DNA mixture were corrected by subtracting the CD signals of complexes between 340 and 220 nm to eliminate their influence.

NMR spectroscopy

The ^1H NMR spectrum were acquired using a Bruker-600 MHz NMR instrument. The lyophilized RNA samples were dissolved in a 25 mM phosphate-buffered saline solution (pH 7.0) with 100 mM K^+ and 10% D_2O, resulting in a final concentration of 0.7 mM per strand.

Stopped-flow

The experiments were conducted using an SX20 Stopped-Flow Spectrometer. To investigate RNA formation dynamics, RNA samples (F-MG-T or F-MG-Mut-T) were mixed with a K^+-containing buffer (10 mM Tris-HCl, 200 mM KCl, pH = 7.2). To explore the kinetics of chiral supramolecular complex on F-MG-T G-quadruplex, the RNA sample (F-MG-T) was mixed with a metallohelices-containing

buffer. Fluorescence changes were monitored at an excitation wavelength of 492 nm and emission wavelength of 578 nm.

Fluorescence melting

The fluorescence melting experiments were conducted using a qTOWER3G Touch instrument (Analytik Jena GmbH, Jena, Germany). Melting curves were obtained by monitoring the change in FAM label emission involved in FRET pair during temperature increase. The melting procedure consisted of a 30-minute incubation at 4 °C followed by a gradual temperature ramp at 1 °C/min with fluorescence measurement at each degree increment.

Thermodynamic parameters for intramolecular RNA G-quadruplex formation, including the enthalpy change (ΔHo), entropy change (ΔSo), and free energy change (ΔGo), were calculated based on the fitting of melting curves to theoretical equations. ΔHo was determined as the slope of ln Ka versus 1/T plot using the equation lnKa = −(ΔHo/RT) + ΔSo/R, where ΔSo was calculated from the y-axis intercept representing entropy change. The standard Gibbs's equation was used to calculate the free energy change (ΔGo25) as ΔGo25 = ΔHo - TΔSo.

Immunofluorescence assays

Cells were fixed with 4% paraformaldehyde and permeabilized with 0.5% Triton X-100-PBS for 15 minutes, followed by blocking with a solution of 3% BSA for 30 minutes. Subsequently, the cells were incubated with the primary antibody at 4°C for 16 hours (Wilton, UK). Fluorescent secondary antibodies were used at a dilution ratio of 1:1000 and incubated at 37°C for 45 minutes. DAPI staining was performed in the dark for two minutes before capturing images using confocal laser-scanning microscopy.

UV Titrations

For UV titrations, a 1.0×10-5 M solution of the supramolecular chiral compound was prepared in buffer (10Mm Tris, 100Mm K^+, Ph=7.2). This solution was placed in a cuvette (2 ml) at 25oC. The sample was then titrated with a solution of the RNA. The maximum change in absorbance was calculated from the theoretical fit at complete 1:1 binding isotherm was assumed for all titrations. The association constant was determined using a nonlinear curve-fitting method based on the following equation:

$A_{nt}=A_0+(((A_{gh}-A_0)/2)/H_0) * (H_0+G_0+1/Ka\text{-sqrt}((H_0+G_0+1/Ka)^{\wedge 2}-4*H_0*G_0))$

Vectors construction and transfection

The reporter vector, pLV-MG-WT-EGFP, was constructed by incorporating the MG into the pLV-EGFP-N backbone (Inovogen Tech. Co., Beijing, China). The MG-WT was point mutated to generate pLV-MG-Mut-EGFP using the Mutagenesis kit (Agilent Technologies, Palo Alto, CA, USA). The transformation of plasmids uses DH5α cells (#AG11806, ACCURATE BIOTECHNOLOGY(HUNAN） CO.,LTD, ChangSha, China), and is activated by heating at 42°C. For transfection, Lipofectamine 2000 (Invitrogen, CA, USA) was used according to the manufacturer's protocol. The transfected cells were harvested after 48 to 72 h.

Western blot assays

The cells were lysed using RIPA buffer (CWBIO, Beijing, China). Following separation by SDS-PAGE, the proteins were transferred onto nitrocellulose filter membrane (NC) membranes and subsequently blocked in a TBST solution containing 5% skim milk. After incubation with antibodies specific for EGFP (#AF0159; Beyotime, China) or β-actin (#K101527P; Beijing Solarbio Science & Technology Co., Ltd., Beijing, China), the blots were probed with anti-mouse (#bs-0296G-HRP; Bioss, Beijing, China) or anti-

rabbit (#bs-80295G-HRP; Bioss, Beijing, China) secondary antibodies. Protein detection was performed using the ECL Chemiluminescence Kit (#KGC4601-100; Keygen BioTECH, China).

Cytotoxicity test (CCK8 method)

Vero, HEK293T, or MCF-7 cells were seeded in 96-well plates and incubated overnight in a 5% CO_2 environment. Different drug concentrations were added to the corresponding medium, and the cells were cultured for 72 hours in the same plates. After incubation, CCK8 reagent was added at a 1:10 ratio as per the manufacturer's protocol and placed in the cell incubator for 1 hours. The absorbance value was measured at a wavelength of 450nm using a microplate reader. Cell activity inhibition rate was calculated using a formula, CC_{50} was determined using Graphpad 9 software, and an inhibition rate curve was fitted.

Fluorescence assays

The EGFP protein expression in cells was visualized using confocal laser scanning microscopy. The ImageJ software was used to normalize the EGFP intensity against its corresponding DAPI intensity.

Real-time PCR assay (qPCR)

The 2X drug solution was prepared using 2% FBS MEM medium. The liquid in the 96-well plate was aspirated, and each drug well received 0.1 mL of 2X drug diluent. Six dilutions were prepared with three replicate wells for each dilution. Dilutions were added sequentially from low to high concentration in a 96-well plate. The negative control wells contained cells without virus, while the virus control wells contained only cells with virus and no drug treatment. The cells were incubated at 37°C in a CO_2 cell incubator for 1-2 hours.

Vaccinia virus TTV-EGFP and monkeypox virus MPXV-B.1-Chinese-C-TAN-CQ01 were diluted in 2% FBS MEM medium to a final concentration of 100 PFU/100 µL. Each well received 100µL of the viral suspension, resulting in a total of 100 PFU per well at 37°C. The cells were cultured in a CO_2 incubator with a concentration of CO_2 maintained at around 5% incubation continued for 72 to 96 hours. The total DNA was extracted using the Tianlong nucleic acid analyzer, and the target genes of monkeypox virus F3L and vaccinia virus E3L were detected by quantitative PCR (qPCR) using 2×Taq Pro U+ Multiple Probe qPCR Mix (Imbio Biotechnology (Beijing) Co., LTD.). The primer and probe sequences are provided in Table 1. The viral copy number was determined based on a standard curve, and the drug inhibition rate was calculated as follows: (viral control copy number - experimental well copy number)/viral positive control copy number ×100%. The EC_{50} value was calculated using Graphpad software, and the inhibition rate curve was fitted accordingly.

Plaque reduction test

The 2%FBS MEM medium was utilized for the preparation of a 2X drug solution. Subsequently, the liquid was aspirated from the 12-well plate and replaced with 0.5mL of 2X drug diluent in each well. A total of six dilutions were prepared, with three duplicate wells for each dilution. The diluents were added to the plate in ascending order of concentration, starting from low concentration to high concentration. Negative cell control consisted of pure cells without virus, while positive virus control comprised pure cells without any drug treatment. Incubation was carried out at a temperature of 37°C and under a CO_2 atmosphere containing 5% for a duration ranging between 1-2 hours.

The vaccinia virus TTV-EGFP and monkeypox virus MPXV-B.1-China-C-tan-CQ01 were diluted using a medium consisting of 2% FBS MEM, resulting in a final concentration of 100PFU/500µL per well. Each well received an addition of 500µL so that it contained exactly 100 PFU virus particles. Cell culture was continued at a temperature of 37°C under an atmosphere containing 5% CO_2 for 72-96 hours.

After that, 1 mL of a 0.1% crystal violet solution and a 4% poly-formaldehyde fixative were added to each well for fixation and staining for 30 minutes. Subsequently, each well was washed once with 1 mL of ddH_2O. Following drying, the plaques were photographed and counted. The drug inhibition rate was calculated as (number of viral control plaques - number of plaques in experimental wells) divided by the number of viral positive control plaques, multiplied by 100%. The EC_{50} value was determined using Graphpad software, and an inhibition rate curve was fitted.

Reverse transcription-quantitative PCR Test (RT-qPCR)

To assess the activation of inflammatory cytokines against the anti-monkeypox virus by the compounds, we conducted a study on the impact of these compounds on anti-monkeypox virus-induced inflammation using a drug concentration of 5μM. The drug treatment procedure followed the pharmacodynamic qPCR method specific to anti-monkeypox virus. For quantification of cytokine mRNA levels, total RNA was extracted from cells using an automated nucleic acid extractor (Tianlong, China). he expression levels of cytokines were determined using the HiScript II One Step qRT-PCR SYBR Green Kit (Vazyme, China) and analyzed with GAPDH as a reference gene using the 2-ΔΔCt method. The primer sequences for cytokine quantification are provided in Table S2.

Statistical analysis

The statistical analyses were performed using GraphPad Prism software. The plaque count and statistics were analyzed using Image J. The data in this study are derived from at least three independent experiments. And, student's t-test was employed for statistical analysis, with significance defined as *p < 0.05; **p <0.01; ***p < 0.001; ****p < 0.0001; n.s., not significant.

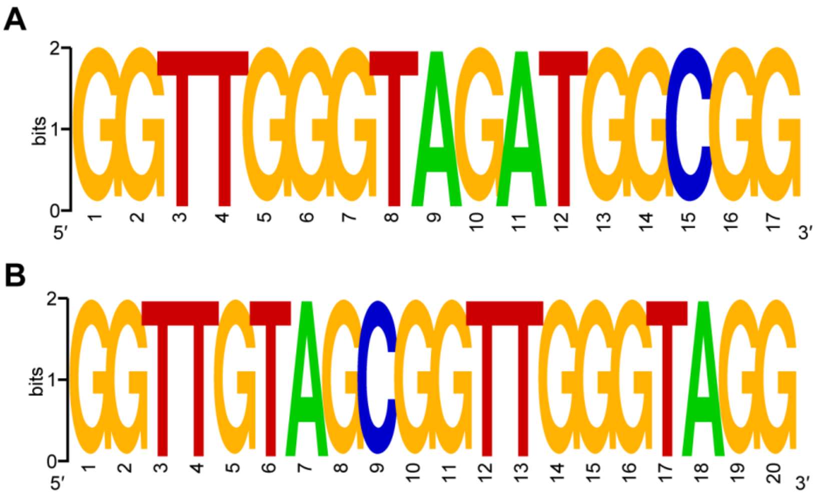

Figure S1. A total of 4771 complete genomic sequences of MPXV genome were retrieved from the National Center for Biotechnology Information (NCBI) (https://www.ncbi.nlm.nih.gov/) and the conservation of the two G4 candidates were aligned using WebLogo software.(A: G1; B: G3)

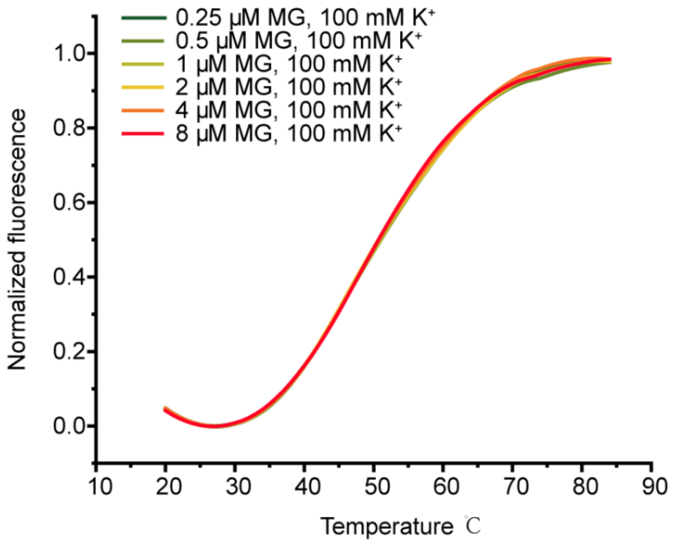

Figure S2. Fluorescence thermal melting curves of MG at different RNA concentrations.

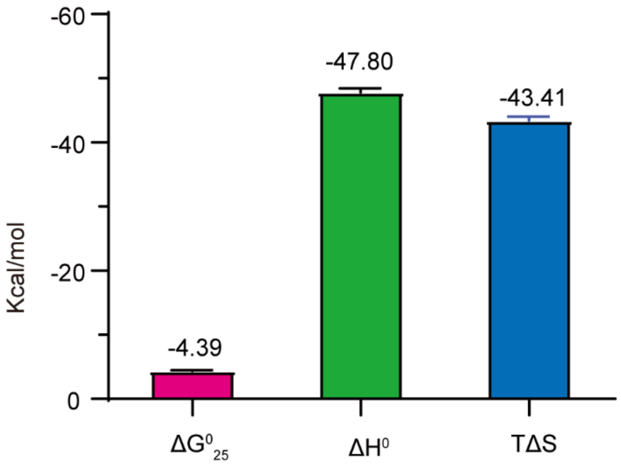

Figure S3. Thermodynamic parameters of MG G4 formation. The results were shown as the means ± SD of three separate experiments.

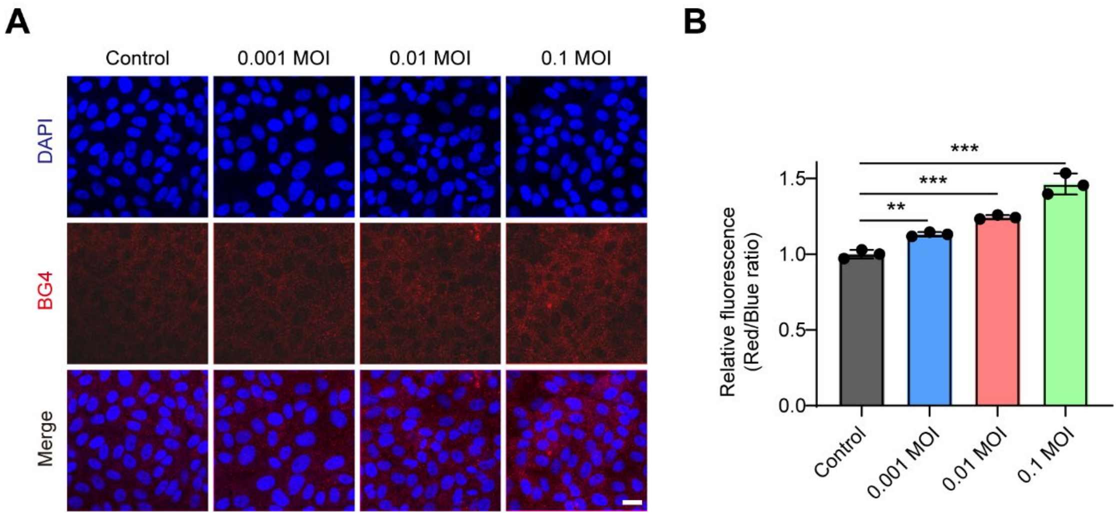

Figure S4. Immunofluorescence assays of MPXV infection in Vero cells. (A) Representative images of immunofluorescence during MPXV infection with different multiplicity of infection. (Bars = 20 μm). (B) Relative fluorescence intensity analyzed by image-J. All the experimental results were analyzed by three independent experiments. **, p<0.01, ***, p<0.001.

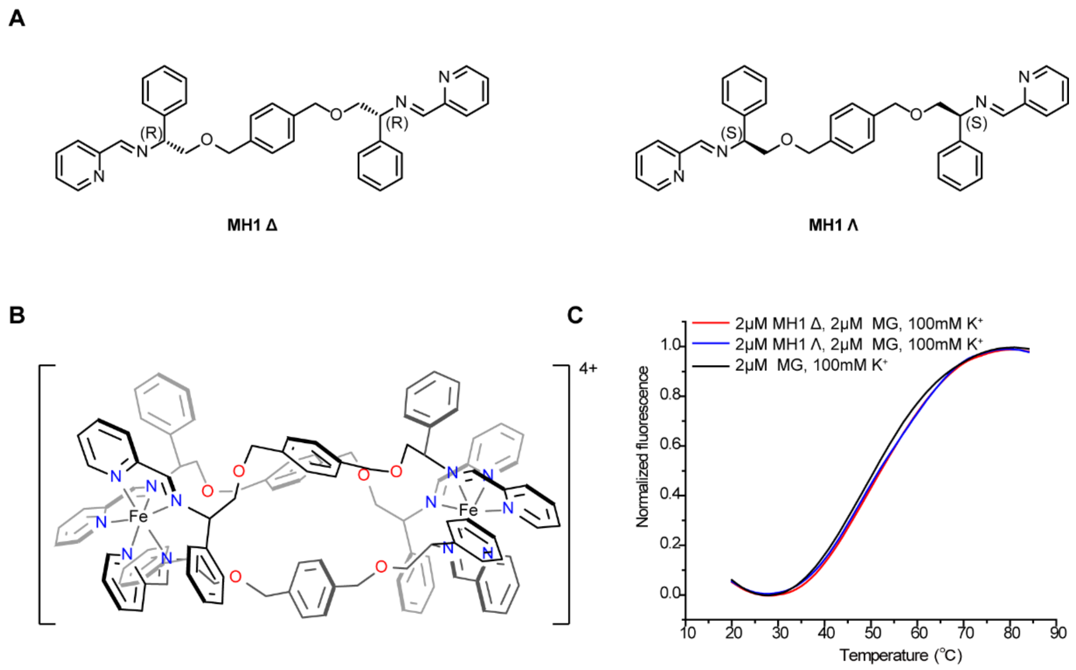

Figure S5. (A) Structures of MH1 Δ and MH1 Λ. (**B**) Structures of the enantiomer cation. (**C**) Fluorescence thermal melting curves of MG G4 (2 μM) in the absence of MH1 Δ or MH1 Λ (2 μM).

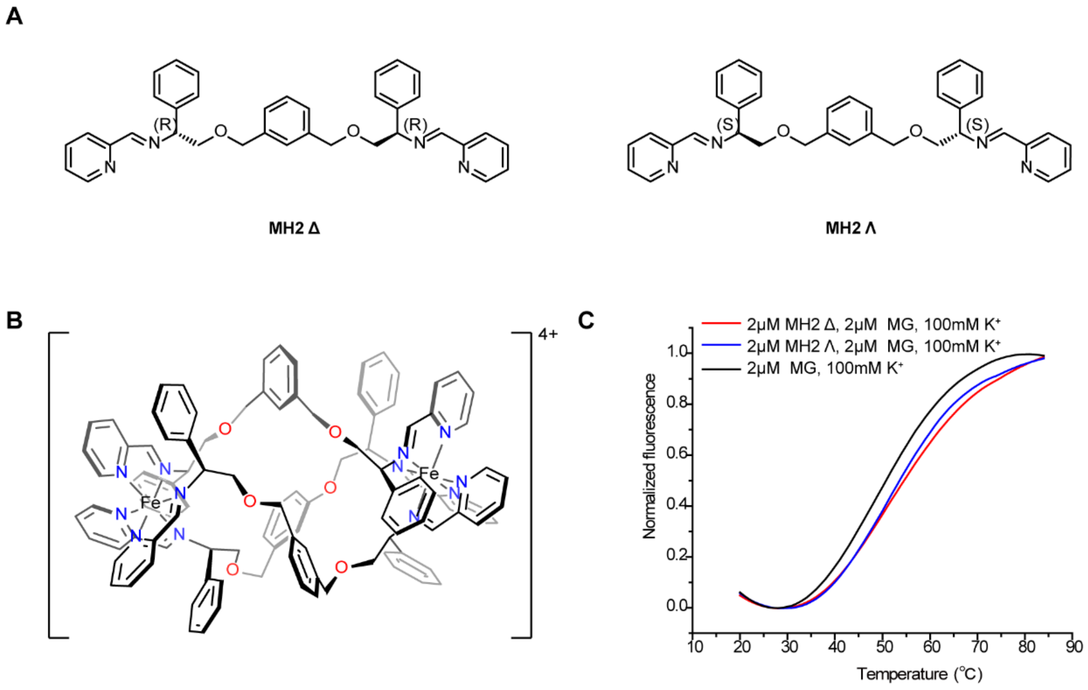

Figure S6. (A) Structures of MH2 Δ and MH2 Λ. (**B**) Structures of the enantiomer cation. (**C**) Fluorescence thermal melting curves of MG G4 (2 μM) in the absence of MH2 Δ or MH2 Λ (2 μM).

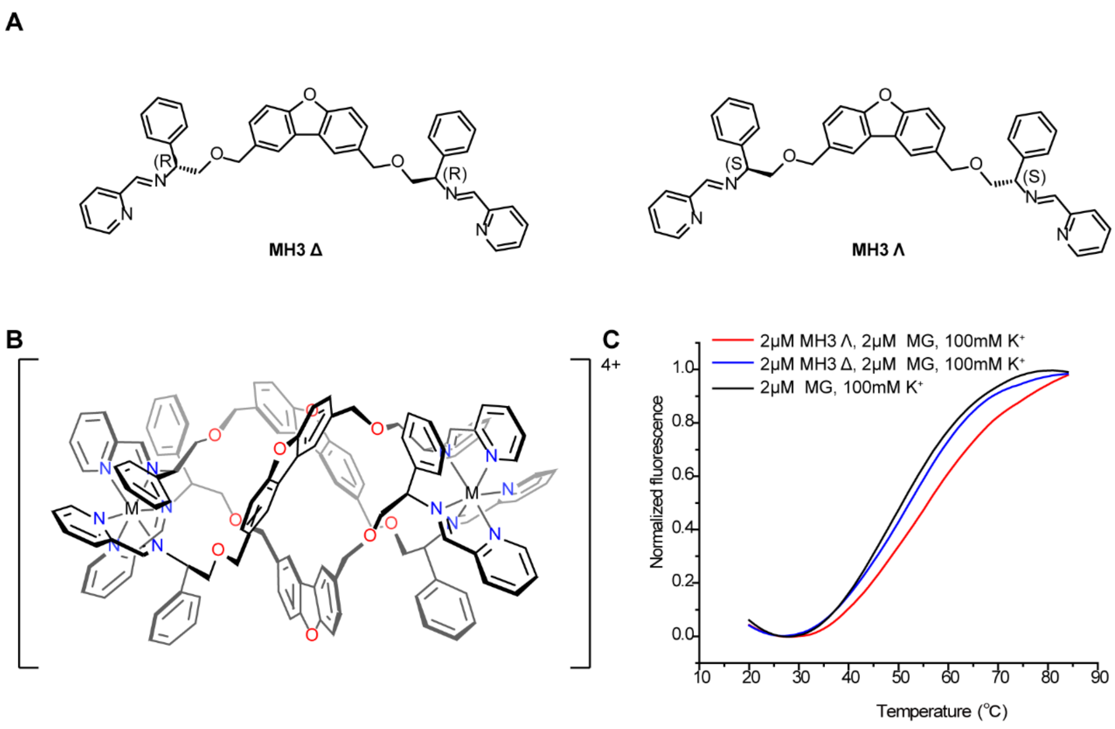

Figure S7. (A) Structures of MH3 Λ and MH3 Δ. (**B**) Structures of the enantiomer cation. (M: Fe^{2+}) (**C**) Fluorescence thermal melting curves of MG G4 (2 μM) in the absence of MH3 Λ or MH3 Δ (2 μM).

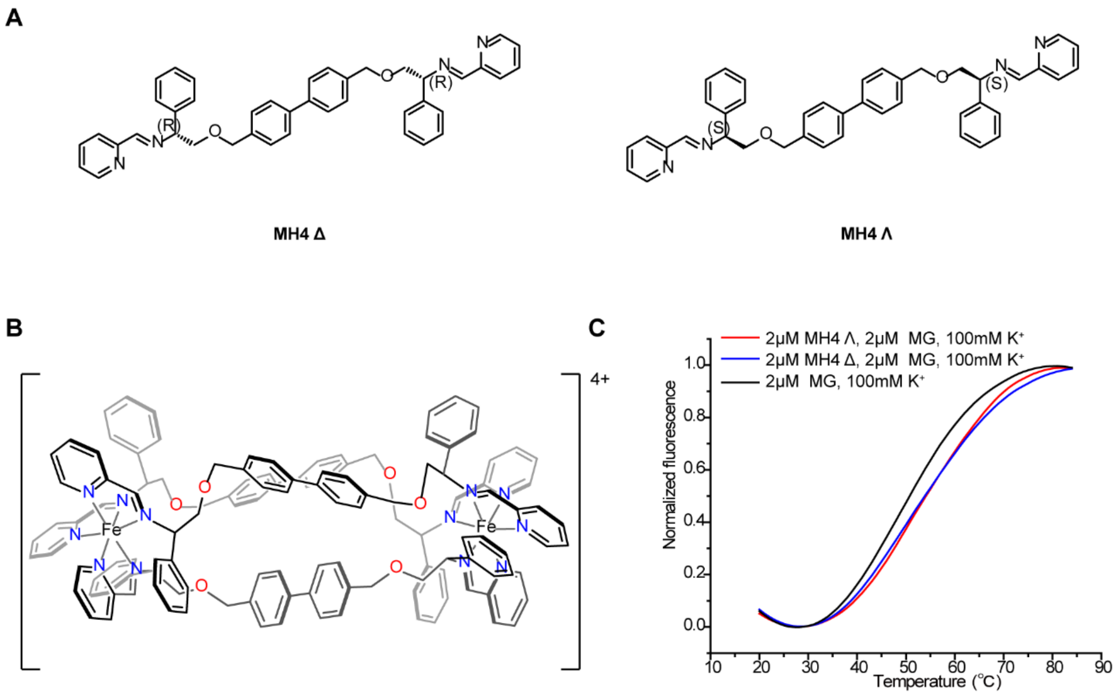

Figure S8. (A) Structures of MH4 Λ and MH4 Δ. (**B**) Structures of the enantiomer cation. (**C**) Fluorescence thermal melting curves of MG G4 (2 μM) in the absence of MH4 Λ or MH4 Δ (2 μM).

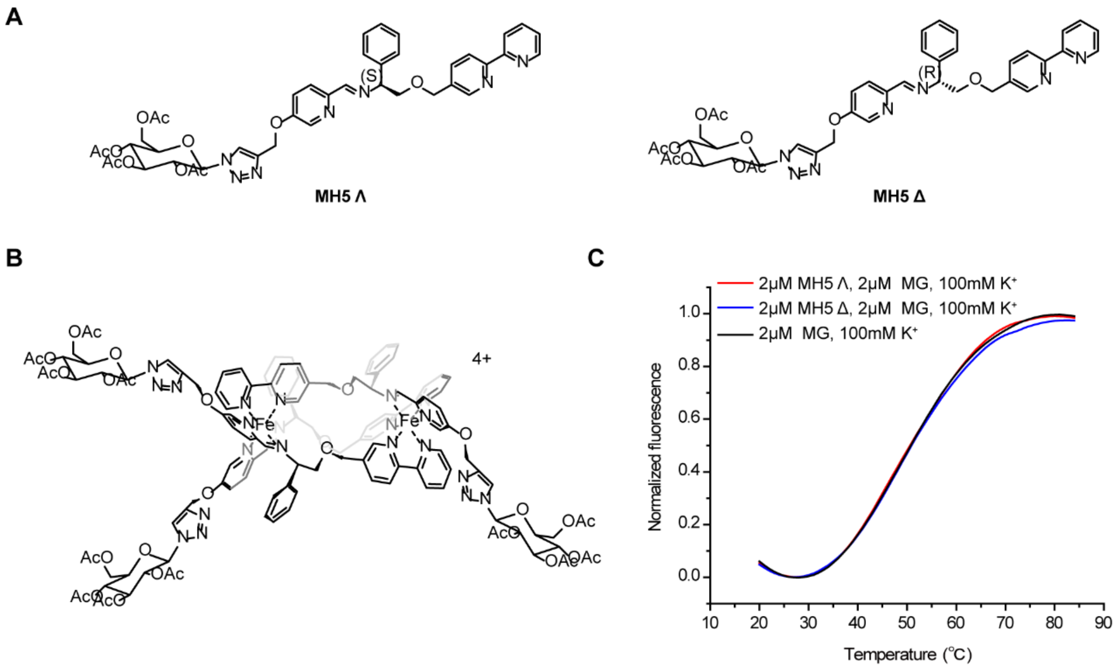

Figure S9. (A) Structures of MH5 Λ and MH5 Δ. (**B**) Structures of the enantiomer cation. (**C**) Fluorescence thermal melting curves of MG G4 (2 μM) in the absence of MH5 Λ or MH5 Δ (2 μM).

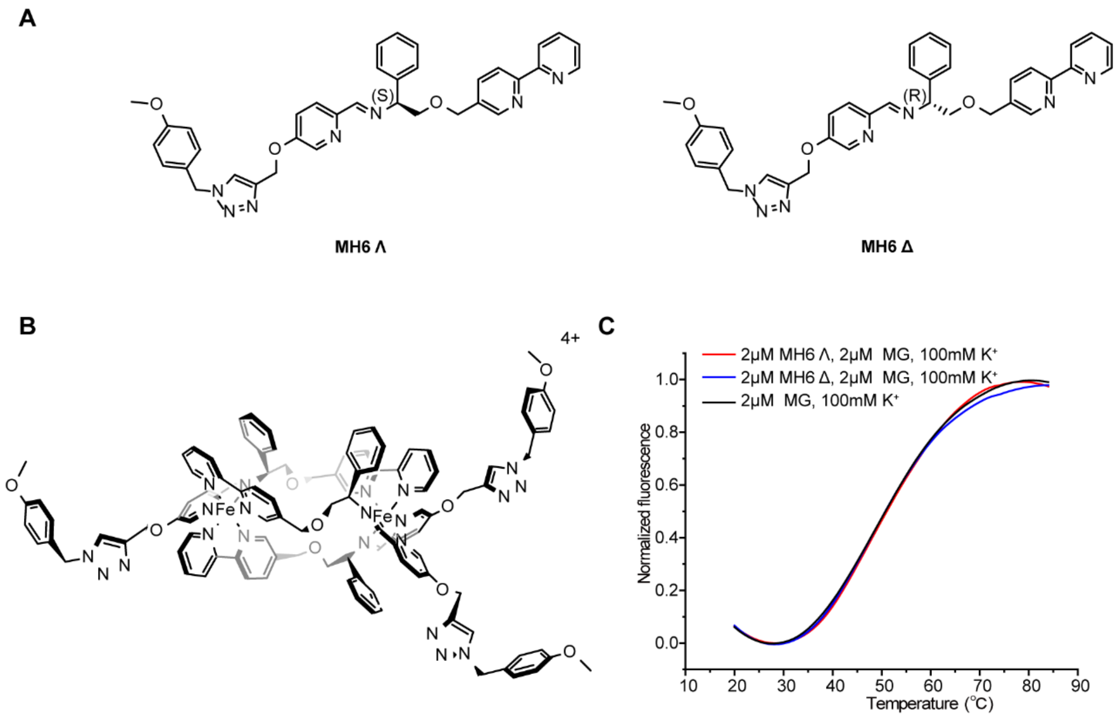

Figure S10. (A) Structures of MH6 Λ and MH6 Δ. (**B**) Structures of the enantiomer cation. (**C**) Fluorescence thermal melting curves of MG G4 (2 μM) in the absence of MH6 Λ or MH6 Δ (2 μM).

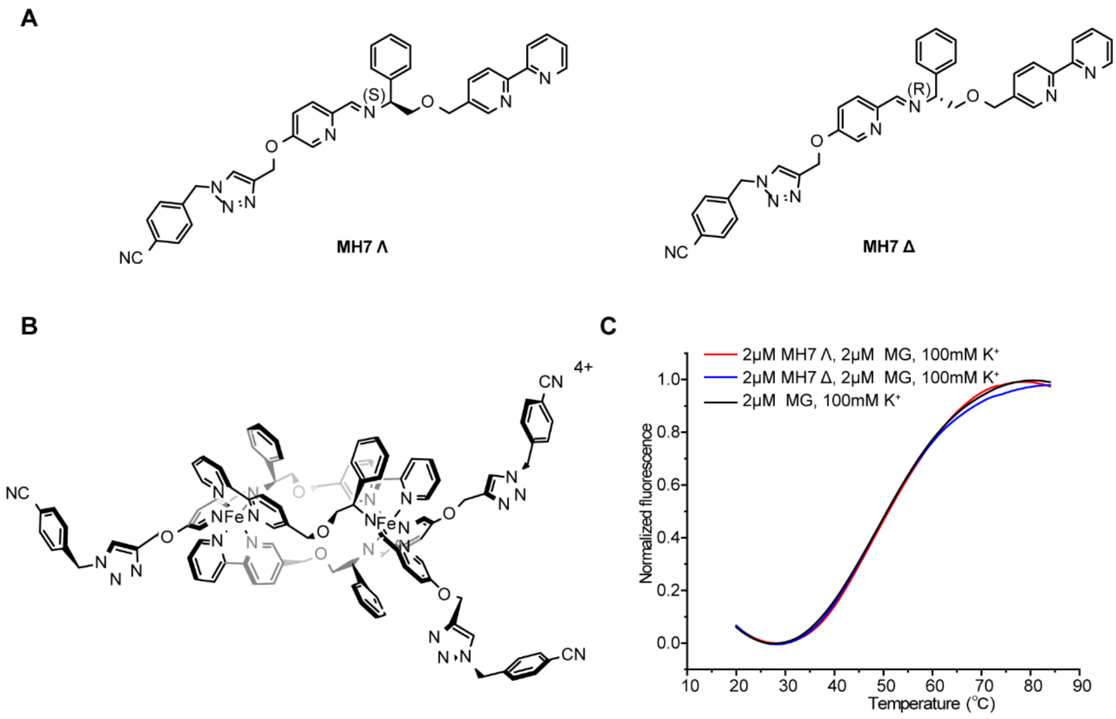

Figure S11. (A) Structures of MH7 Λ and MH7 Δ. (**B**) Structures of the enantiomer cation. (**C**) Fluorescence thermal melting curves of MG G4 (2 μM) in the absence of MH7 Λ or MH7 Δ (2 μM).

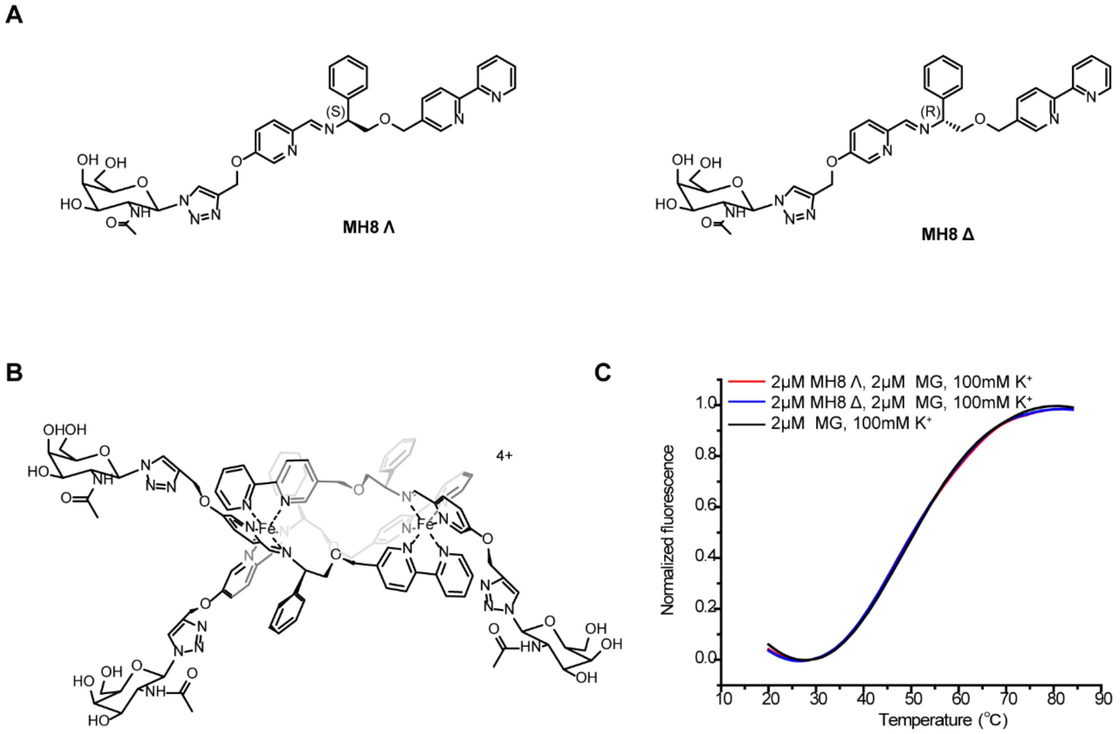

Figure S12. (A) Structures of MH8 Λ and MH8 Δ. (**B**) Structures of the enantiomer cation. (**C**) Fluorescence thermal melting curves of MG G4 (2 μM) in the absence of MH8 Λ or MH8 Δ (2 μM).

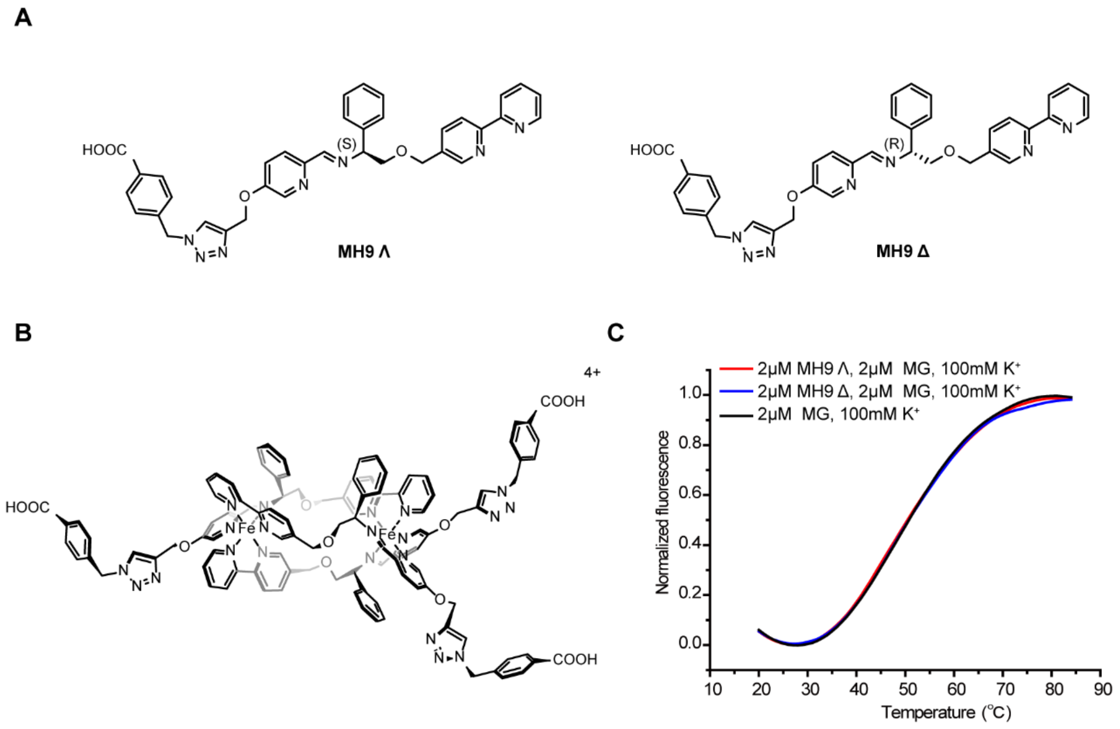

Figure S13. (A) Structures of MH9 Λ and MH9 Δ. (**B**) Structures of the enantiomer cation. (**C**) Fluorescence thermal melting curves of MG G4 (2 μM) in the absence of MH9 Λ or MH9 Δ (2 μM).

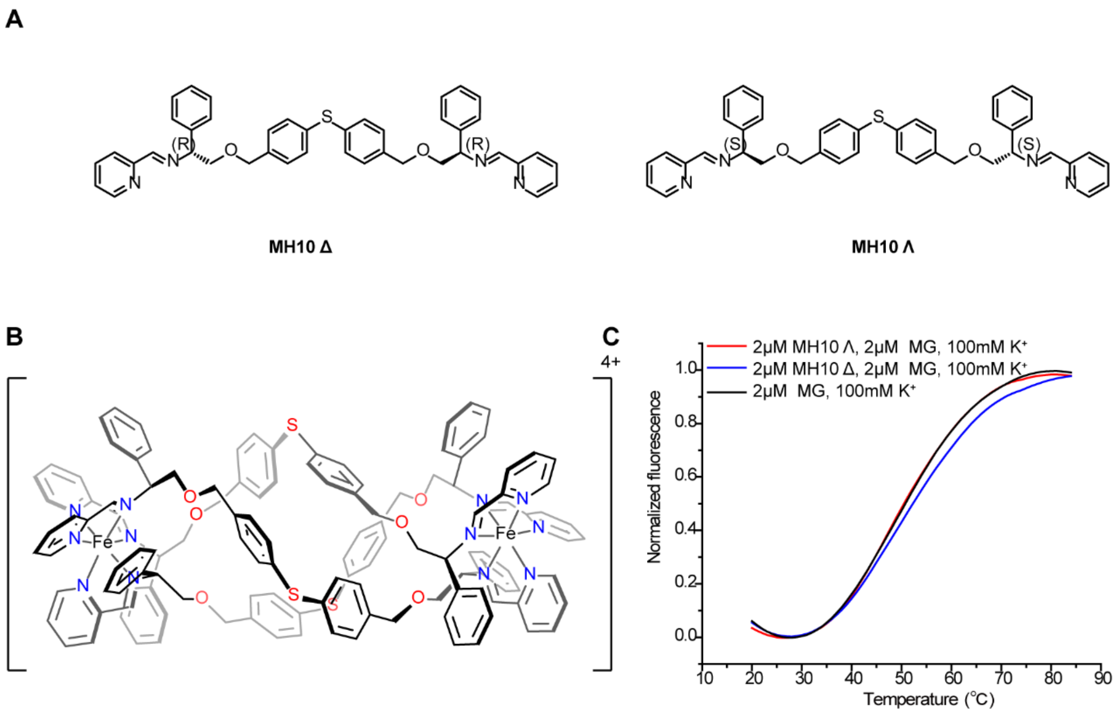

Figure S14. (A) Structures of MH10 Λ and MH10 Δ. (**B**) Structures of the enantiomer cation. (**C**) Fluorescence thermal melting curves of MG G4 (2 μM) in the absence of MH10 Λ or MH10 Δ (2 μM).

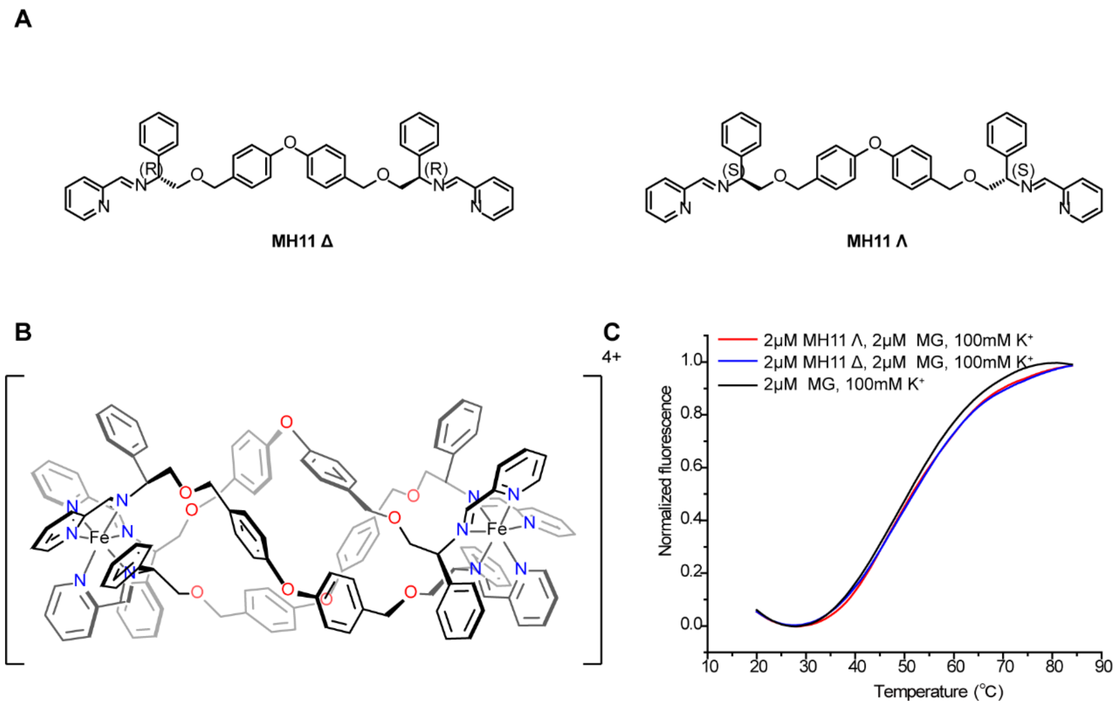

Figure S15. (A) Structures of MH11 Λ and MH11 Δ. (**B**) Structures of the enantiomer cation. (**C**) Fluorescence thermal melting curves of MG G4 (2 μM) in the absence of MH11 Λ or MH11 Δ (2 μM).

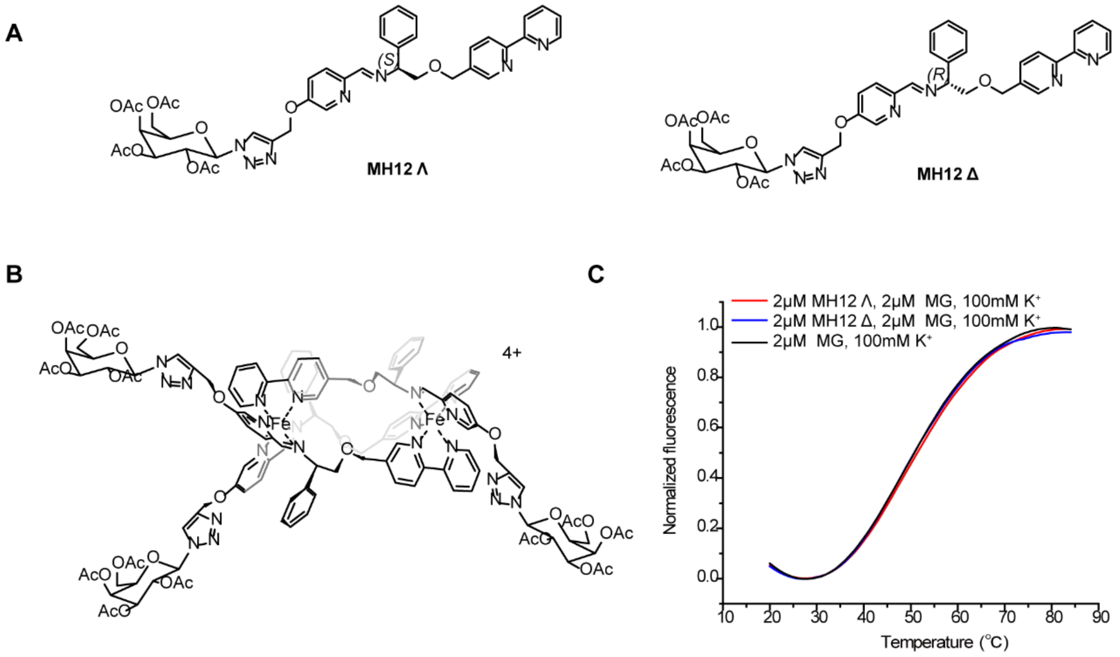

Figure S16. (A) Structures of MH12 Λ and MH12 Δ. (**B**) Structures of the enantiomer cation. (**C**) Fluorescence thermal melting curves of MG G4 (2 μM) in the absence of MH12 Λ or MH12 Δ (2 μM).

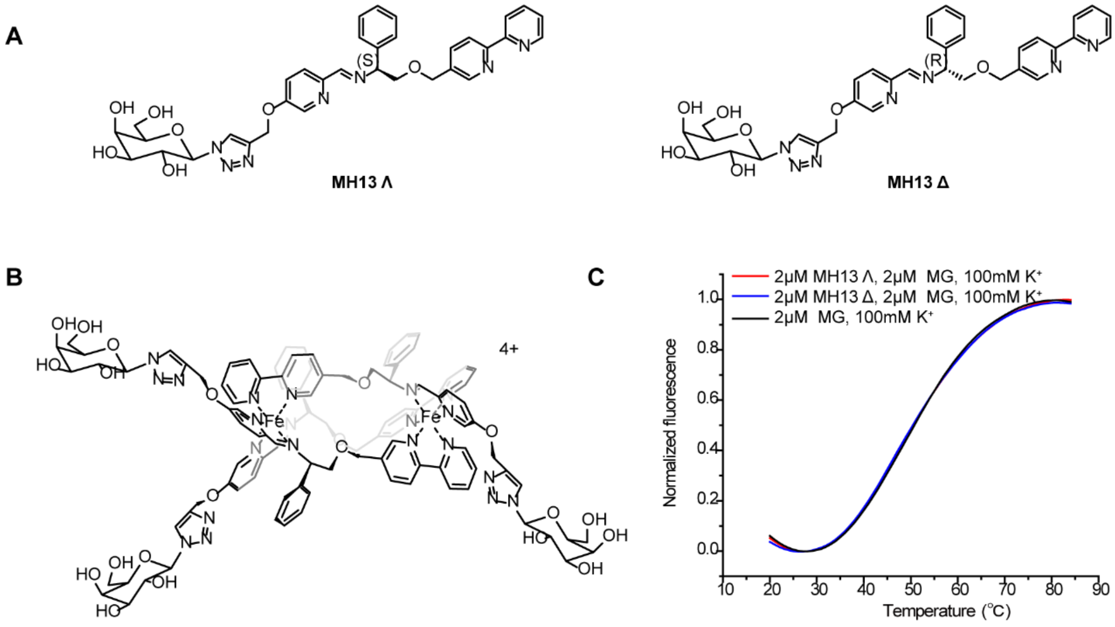

Figure S17. (A) Structures of MH13 Λ and MH13 Δ. (**B**) Structures of the enantiomer cation. (**C**) Fluorescence thermal melting curves of MG G4 (2 μM) in the absence of MH13 Λ or MH13 Δ (2 μM).

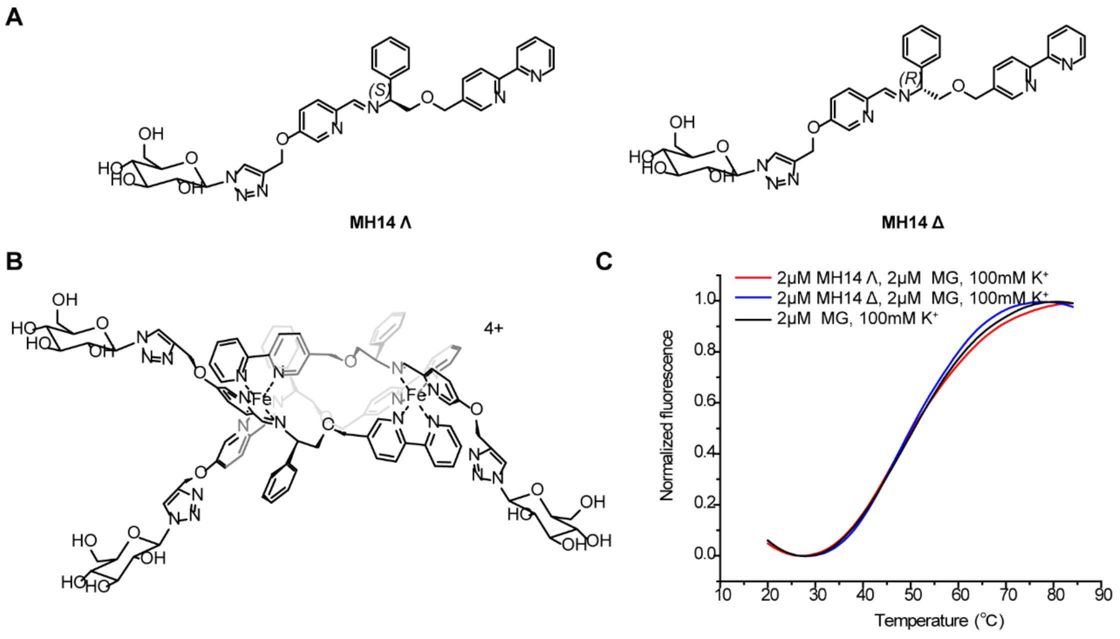

Figure S18. (A) Structures of MH14 Λ and MH14 Δ. (**B**) Structures of the enantiomer cation. (**C**) Fluorescence thermal melting curves of MG G4 (2 μM) in the absence of MH14 Λ or MH14 Δ (2 μM).

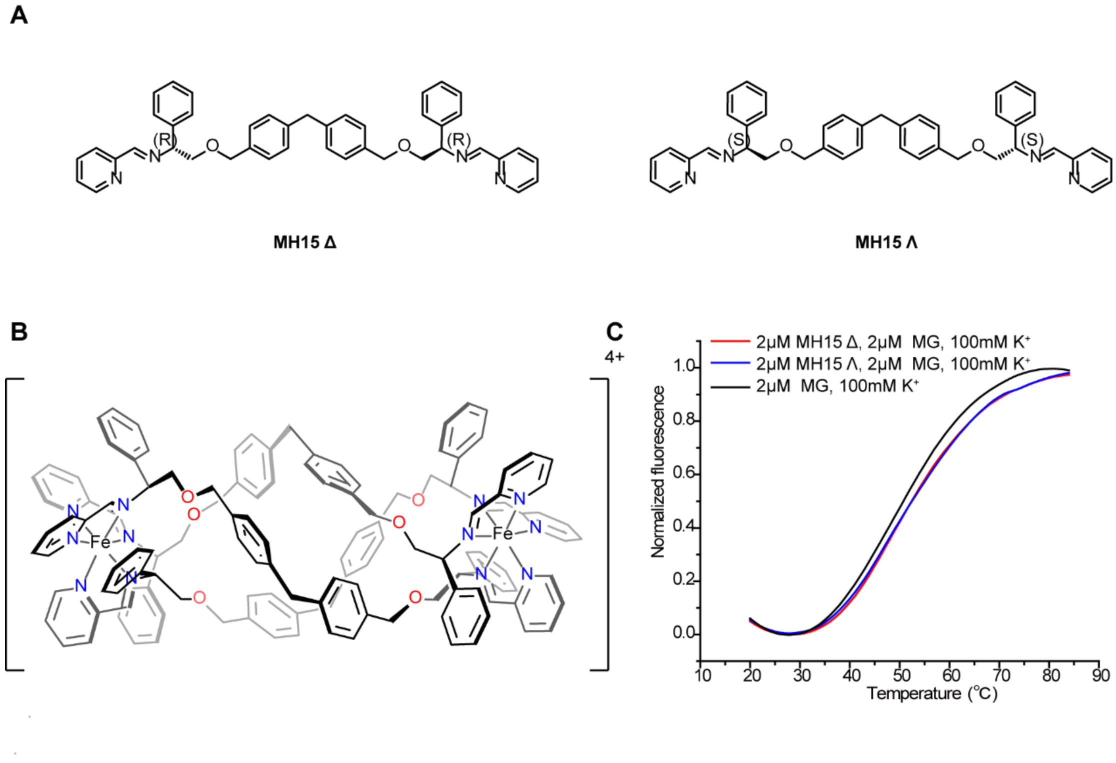

Figure S19. (A) Structures of MH15 Λ and MH15 Δ. (**B**) Structures of the enantiomer cation. (**C**) Fluorescence thermal melting curves of MG G4 (2 μM) in the absence of MH15 Λ or MH15 Δ (2 μM).

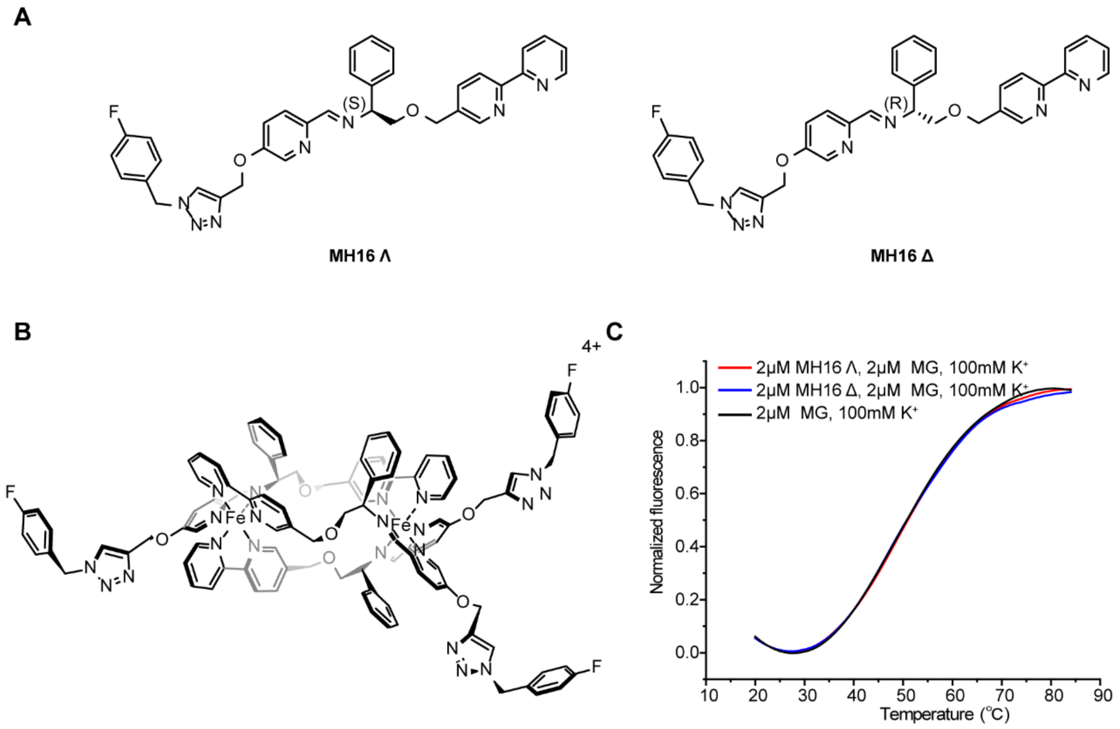

Figure S20. (A) Structures of MH16 Λ and MH16 Δ. (**B**) Structures of the enantiomer cation. (**C**) Fluorescence thermal melting curves of MG G4 (2 μM) in the absence of MH16 Λ or MH16 Δ (2 μM).

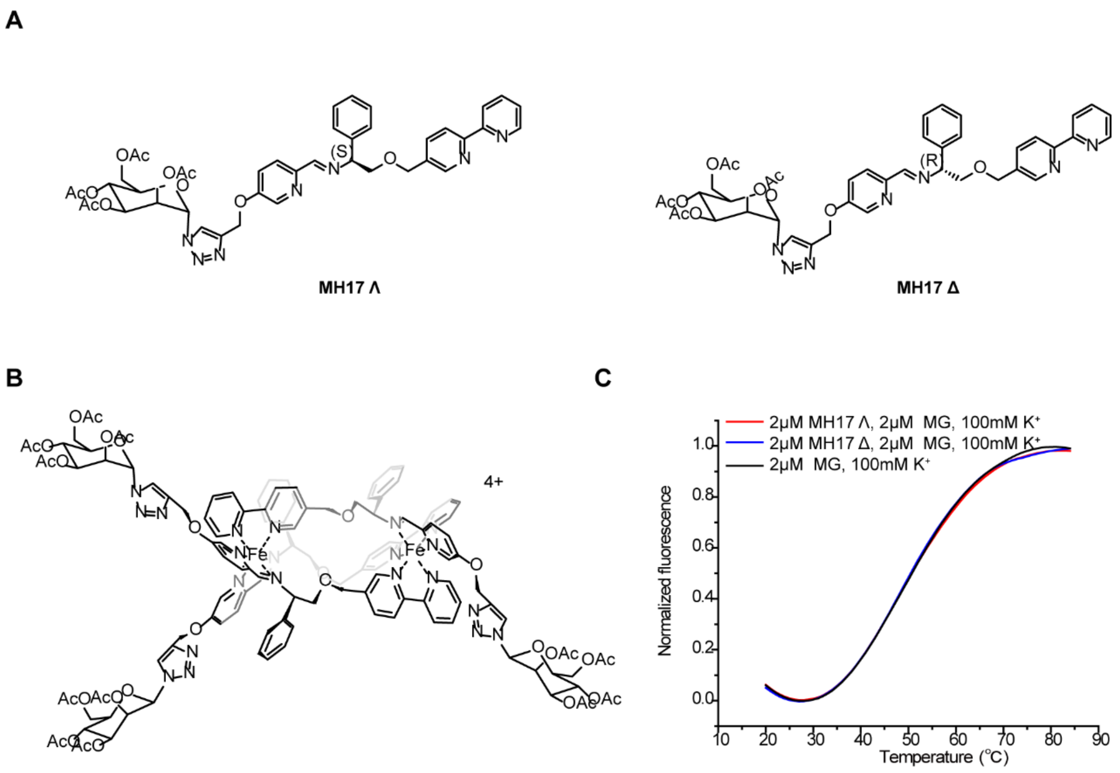

Figure S21. (A) Structures of MH17 Λ and MH17 Δ. (**B**) Structures of the enantiomer cation. (**C**) Fluorescence thermal melting curves of MG G4 (2 μM) in the absence of MH17 Λ or MH17 Δ (2 μM).

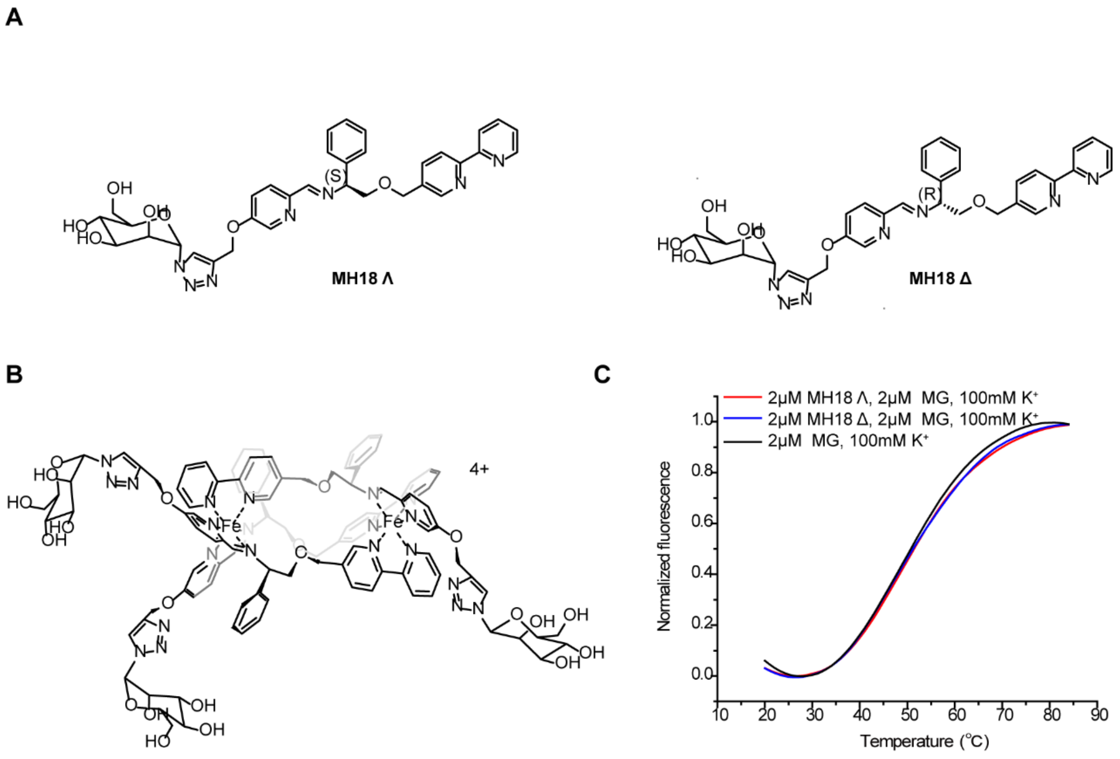

Figure S22. (A) Structures of MH18 Λ and MH18 Δ. (**B**) Structures of the enantiomer cation. (**C**) Fluorescence thermal melting curves of MG G4 (2 μM) in the absence of MH18 Λ or MH18 Δ (2 μM).

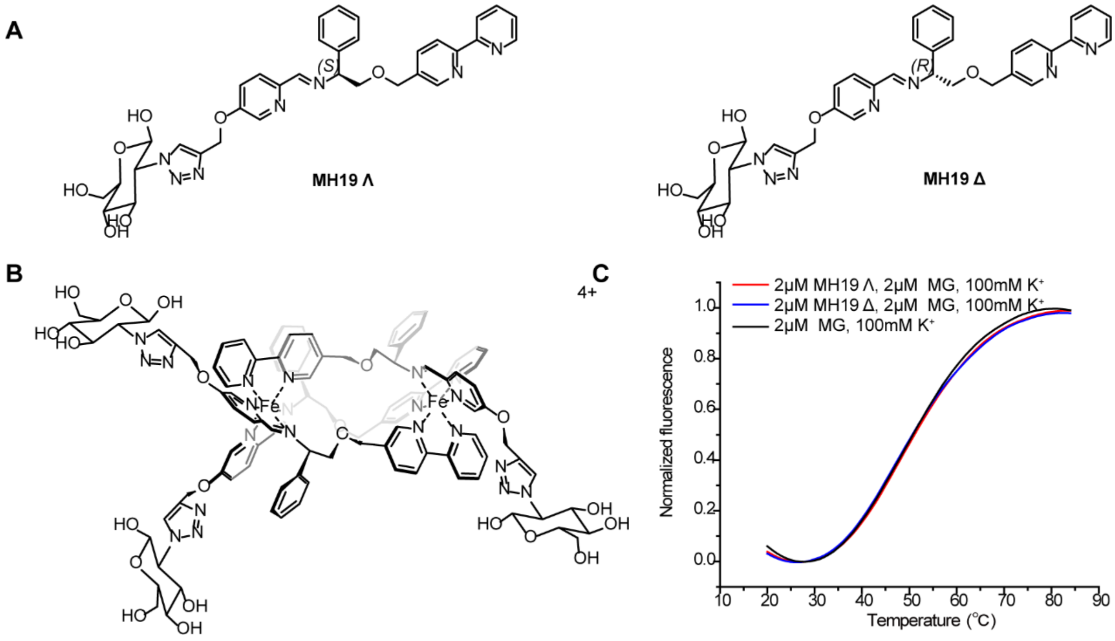

Figure S23. (A) Structures of MH19 Λ and MH19 Δ. (**B**) Structures of the enantiomer cation. (**C**) Fluorescence thermal melting curves of MG G4 (2 μM) in the absence of MH19 Λ or MH19 Δ (2 μM).

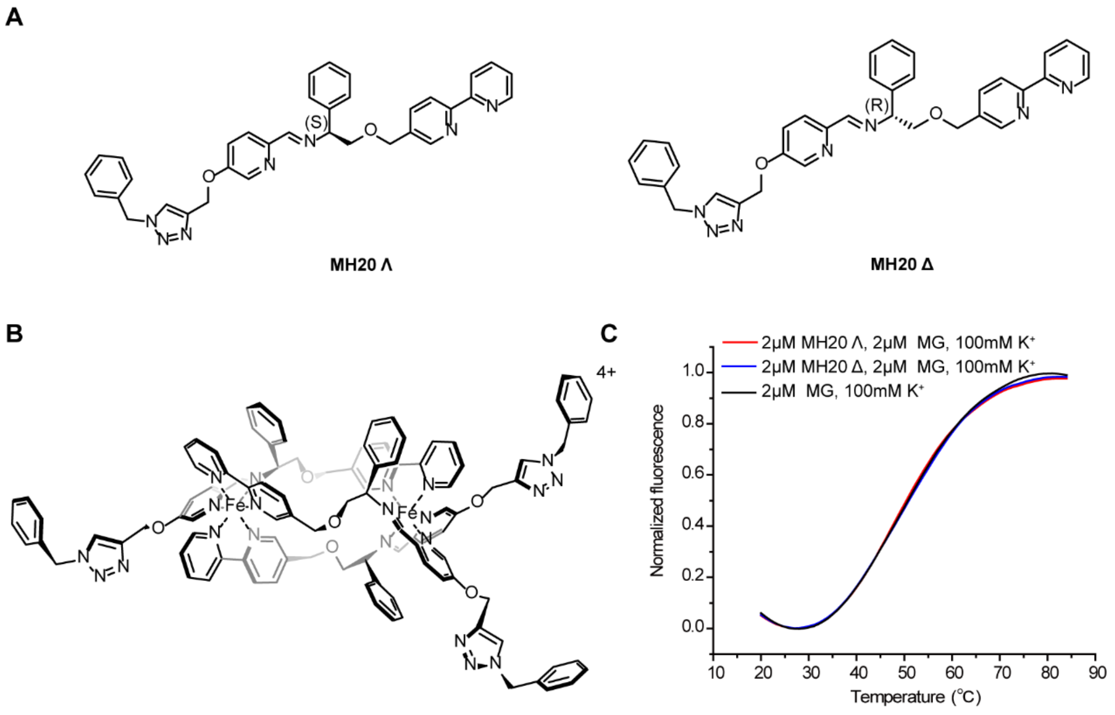

Figure S24. (A) Structures of MH20 Λ and MH20 Δ. (**B**) Structures of the enantiomer cation. (**C**) Fluorescence thermal melting curves of MG G4 (2 μM) in the absence of MH20 Λ or MH20 Δ (2 μM).

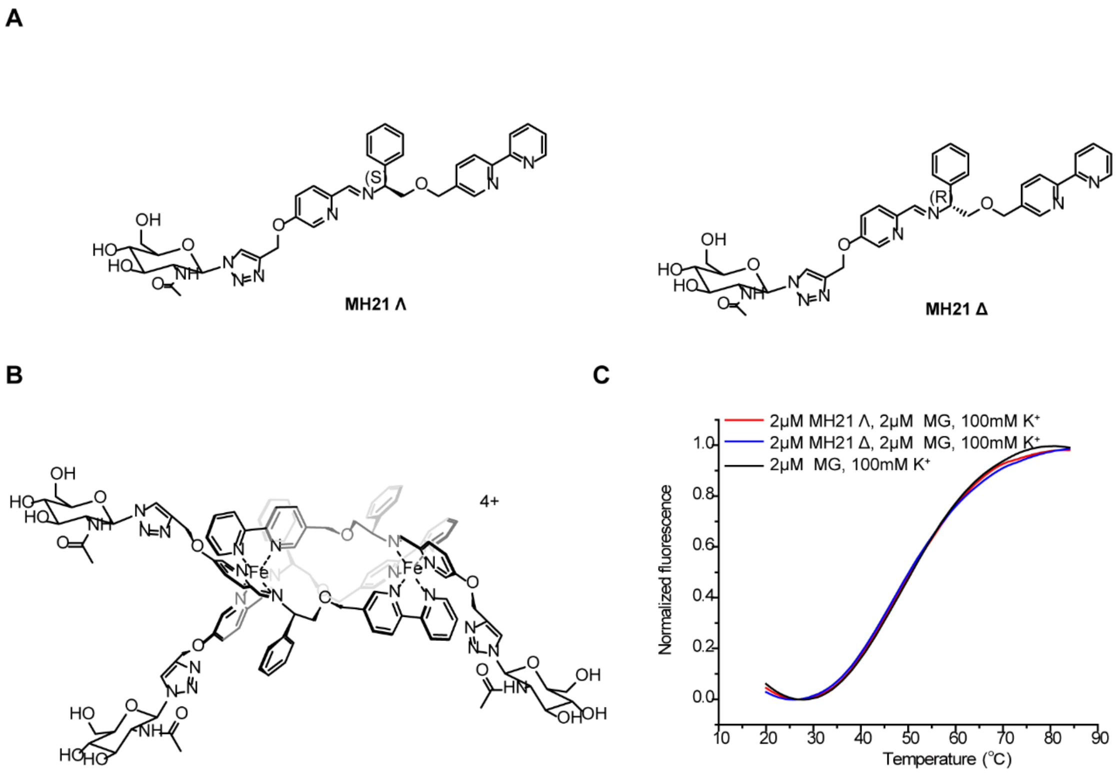

Figure S25. (A) Structures of MH21 Λ and MH21 Δ. (**B**) Structures of the enantiomer cation. (**C**) Fluorescence thermal melting curves of MG G4 (2 μM) in the absence of MH21 Λ or MH21 Δ (2 μM).

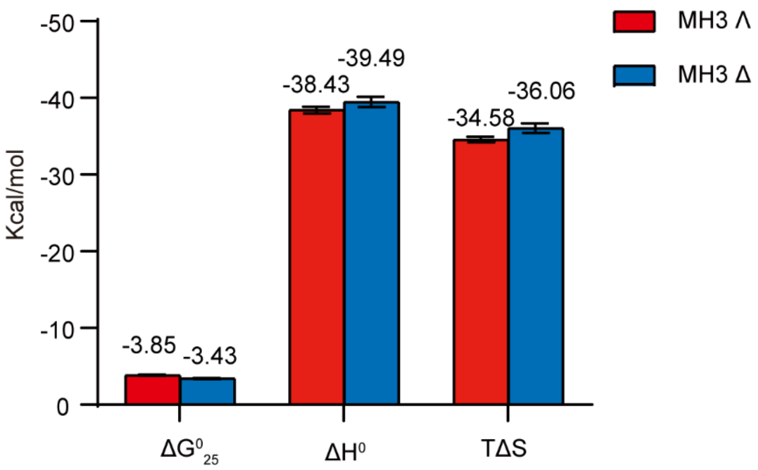

Figure S26. Thermodynamic parameters of the reaction between MH3 and MG. The results were shown as the means ± SD of three separate experiments.

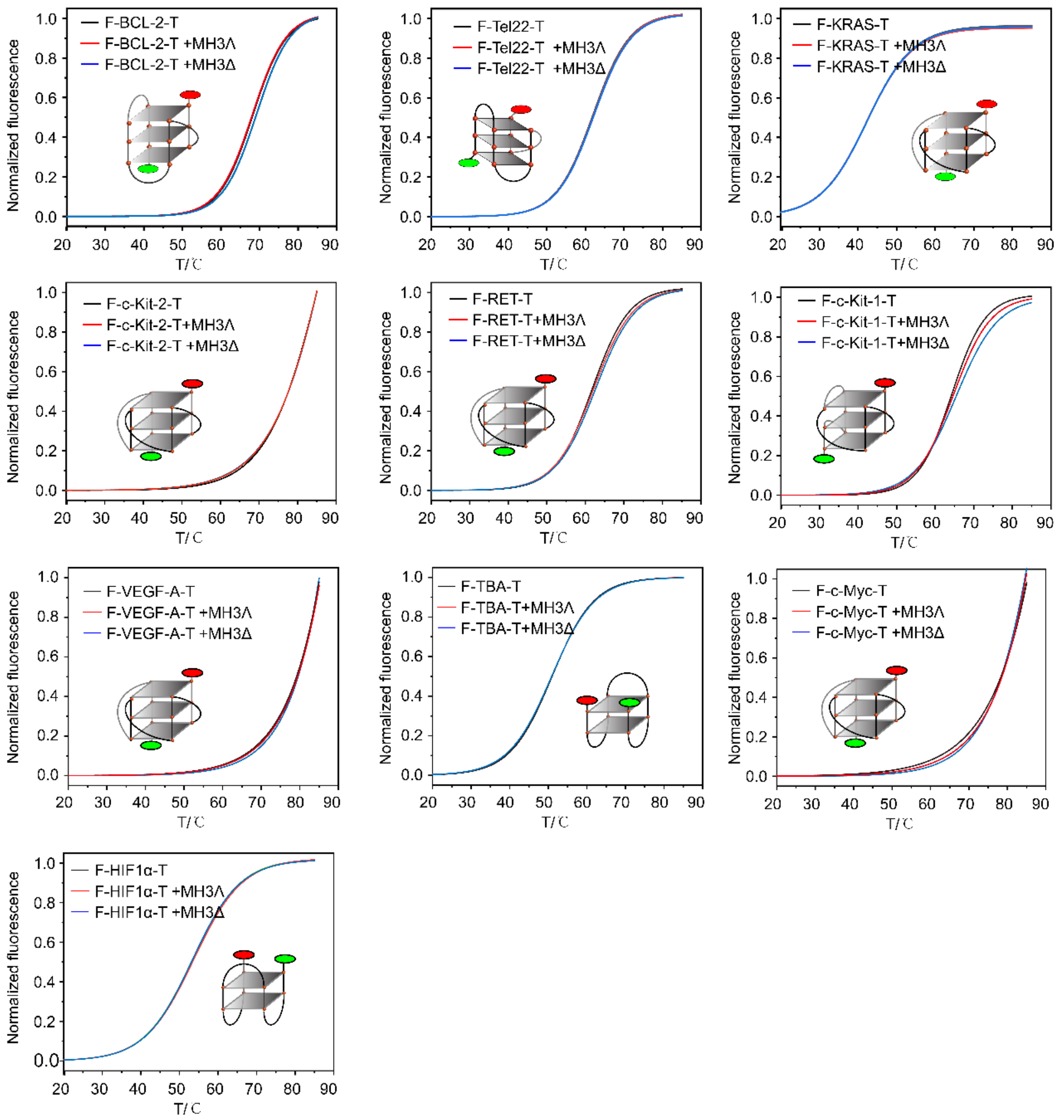

Figure S27. The stabilization effect of MH3 Λ and MH3 Δ on a range of different DNA G-quadruplexes as indicated in the figure. The assays were carried in 10 mM Tris-HCl, 100 mM KCl, pH = 7.2 buffer.

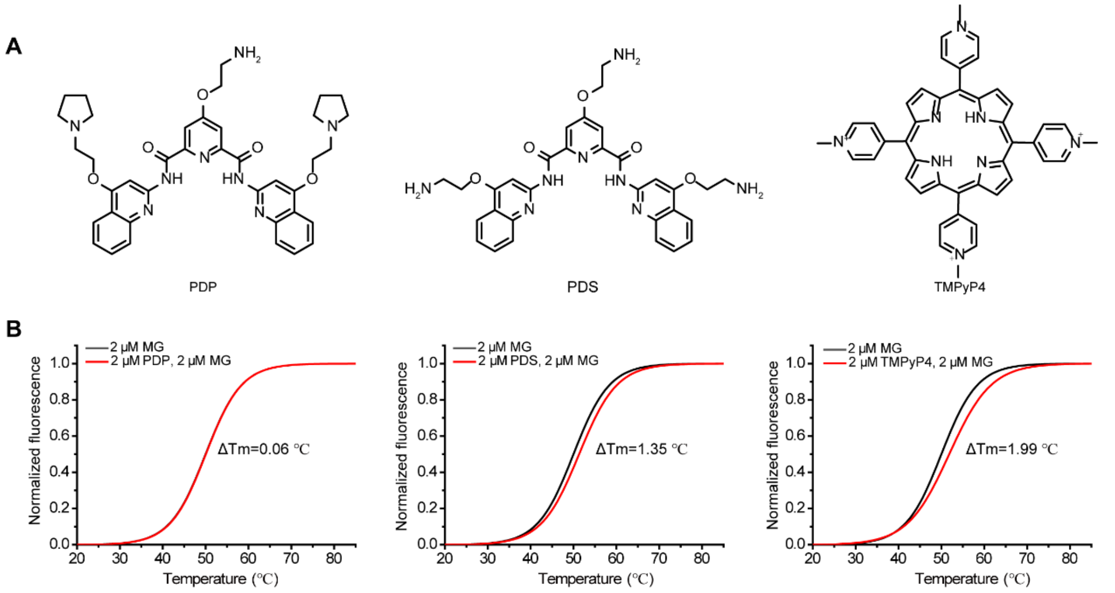

Figure S28. (A) Structures of the G4 ligands. (PDP, PDS and TMPyP4) (**B**) Fluorescence thermal melting curves of MG G4 (2 μM) in the absence of G4 ligands. (PDP, PDS and TMPyP4).

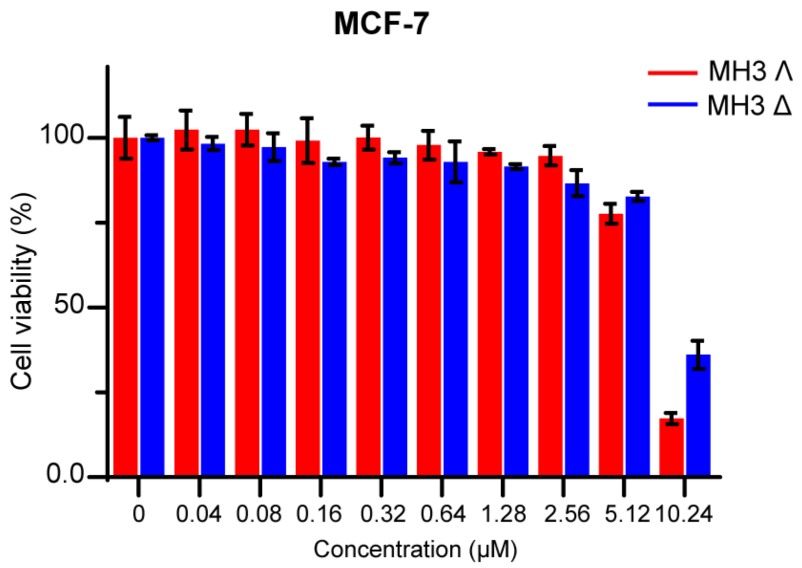

Figure S29. CCK8 assay of MCF-7 cultured with different concentrations of MH3 Λ or MH3 Δ. The MCF-7 cells were treated with various concentrations of MH3 Λ and MH3 Δ (0.16, 0.32, 0.64, 1.28, 2.56, 5.12 and 10.24 μM) for 48h. The results were shown as the means ± SD of four separate experiments.

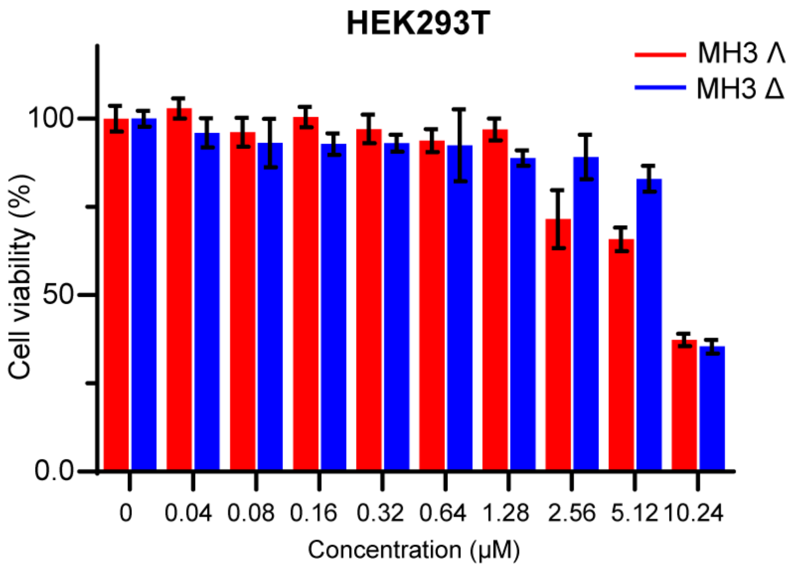

Figure S30. CCK8 assay of HEK293T cultured with different concentrations of MH3 Λ or MH3 Δ. The HEK293T cells were treated with various concentrations of MH3 Λ and MH3 Δ (0.16, 0.32, 0.64, 1.28, 2.56, 5.12 and 10.24 μM) for 48h. The results were shown as the means ± SD of four separate experiments.

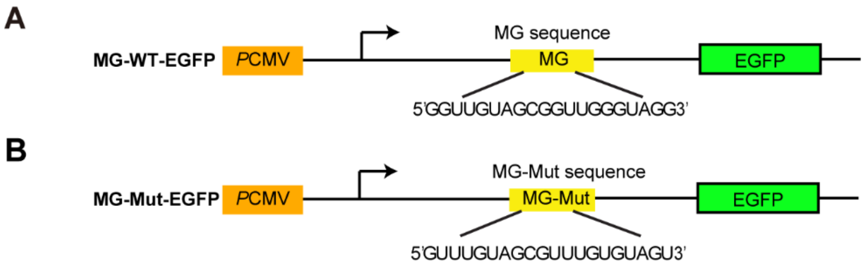

Figure S31 The schematic diagram of the constructed EGFP reporter gene plasmids. (A: MG-WT-EGFP; B: MG-Mut-EGFP)

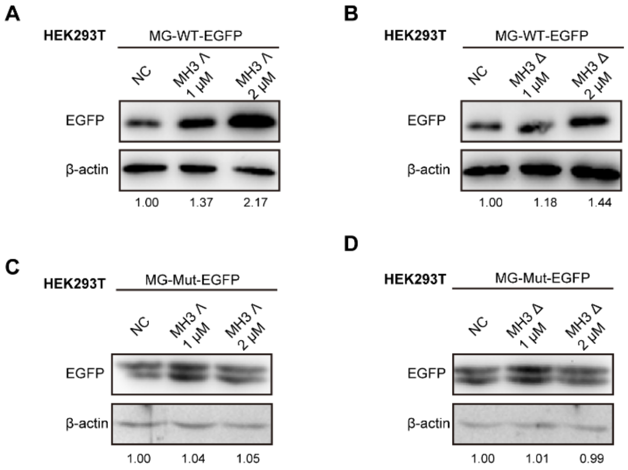

Figure S32. Effect of different concentration MH3 Λ or MH3 Δ on the expression of EGFP reporter by western blot assays (A, B: MG-WT-EGFP; C, D: MG-Mut-EGFP).

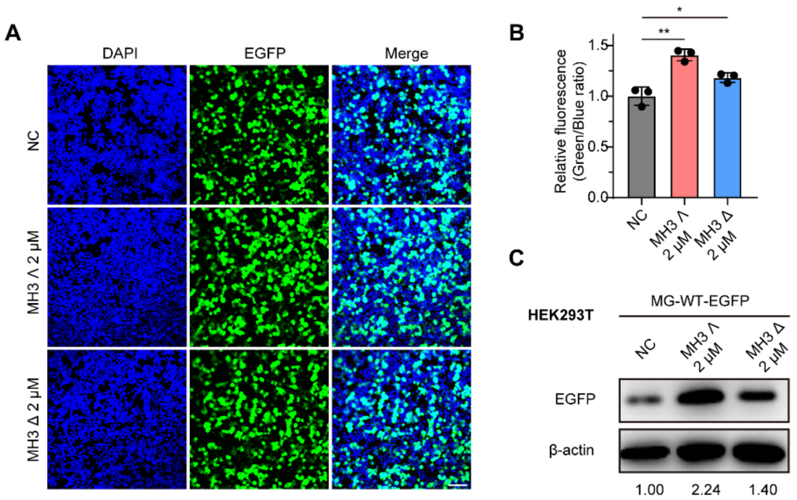

Figure S33. Effects of MH3 Λ or MH3 Δ on the expression of MG-WT-EGFP reporter. (A: Confocal pictures; B: Relative fluorescence intensity analyzed by image-J; C: western blot assay). All the experimental results were analyzed by three independent experiments, error bars represent S.E.M. SEM, standard error of mean significance was analyzed by t-test and scale bar was 100 μm. (*p < 0.05; **p <0.01; Bars=100μM.)

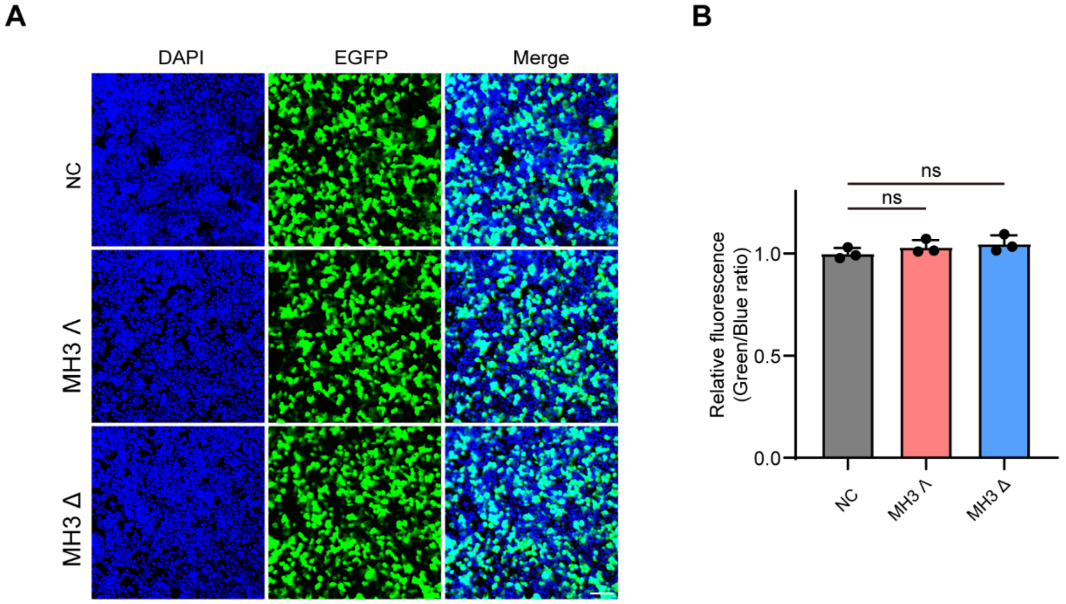

Figure S34. Effects of MH3 Λ or MH3 Δ on the expression of MG-Mut-EGFP reporter. (A: Confocal pictures; B: Relative fluorescence intensity analyzed by image-J). All the experimental results were analyzed by three independent experiments, error bars represent S.E.M. SEM, standard error of mean significance was analyzed by t-test and scale bar was 100 µm. ns, no significant difference.

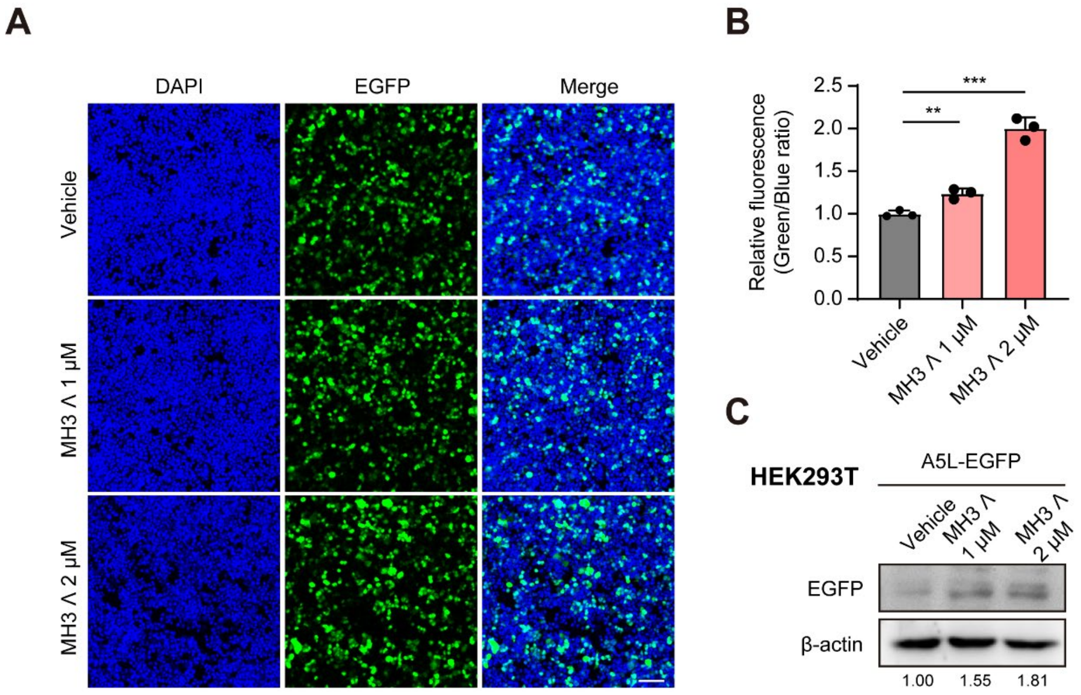

Figure S35. Effects of different concentration MH3 Λ on the expression of A5L-EGFP reporter. (A: Confocal pictures; B: Relative fluorescence intensity analyzed by image-J; C: western blot assay). All the experimental results were analyzed by three independent experiments, error bars represent S.E.M. SEM, standard error of mean significance was analyzed by t-test and scale bar was 100 µm. (**, *p*<0.01; ***, *p*<0.001; Bars=100µm).

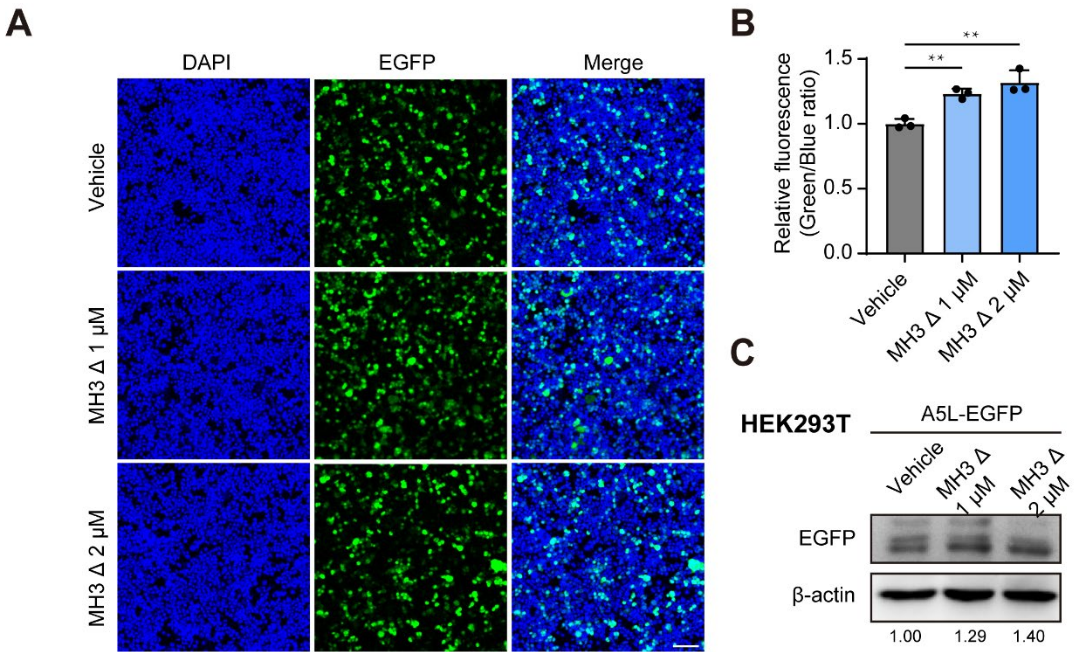

Figure S36. Effects of different concentration MH3 Δ on the expression of A5L-EGFP reporter. (A: Confocal pictures; B: Relative fluorescence intensity analyzed by image-J; C: western blot assay). All the experimental results were analyzed by three independent experiments, error bars represent S.E.M. SEM, standard error of mean significance was analyzed by t-test and scale bar was 100 μm. **, *p*<0.01.

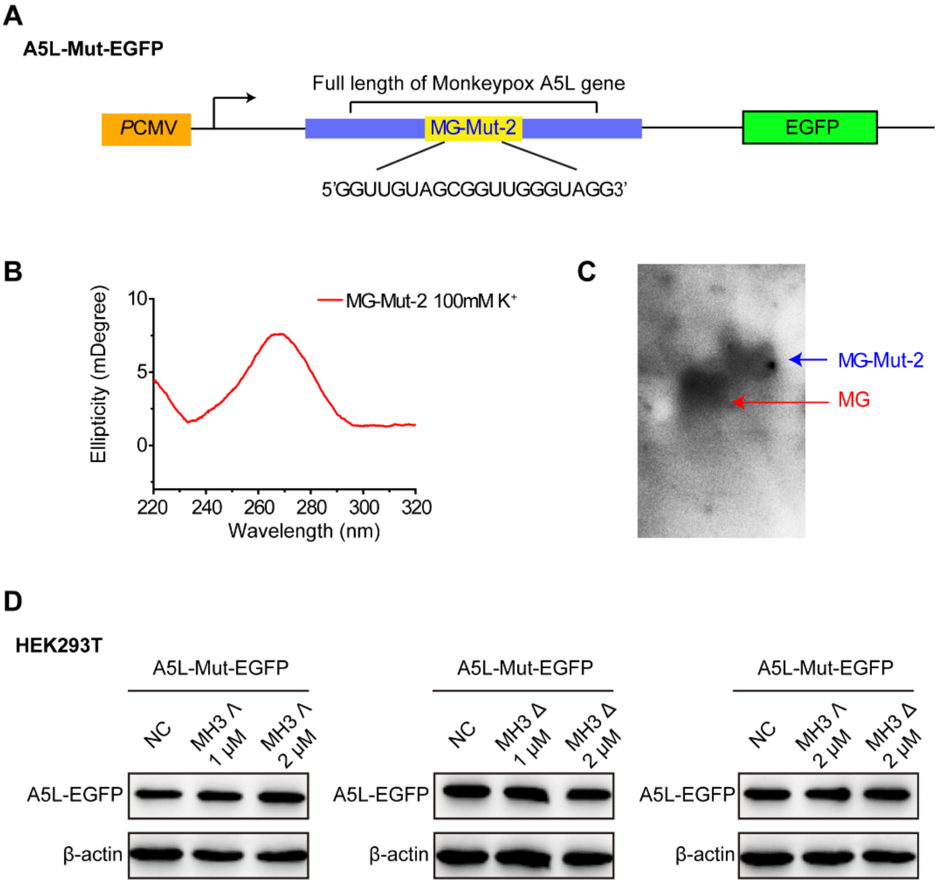

Figure S37. Effects of MH3 Λ or MH3 Δ on A5L-EGFP expression in cells transfected with A5L-Mut-EGFP reporter. (A) The schematic diagram of the constructed A5L-Mut-EGFP reporter gene plasmids. (B) CD spectra of MG-Mut-2. (C) Natural gel electrophoresis Analysis of MG and MG-Mut-2. (D) The effect of MH3 Λ or MH3 Δ addition on A5L-EGFP expression in cells transfected with A5L-Mut-EGFP reporter analyzed by western blot assay.

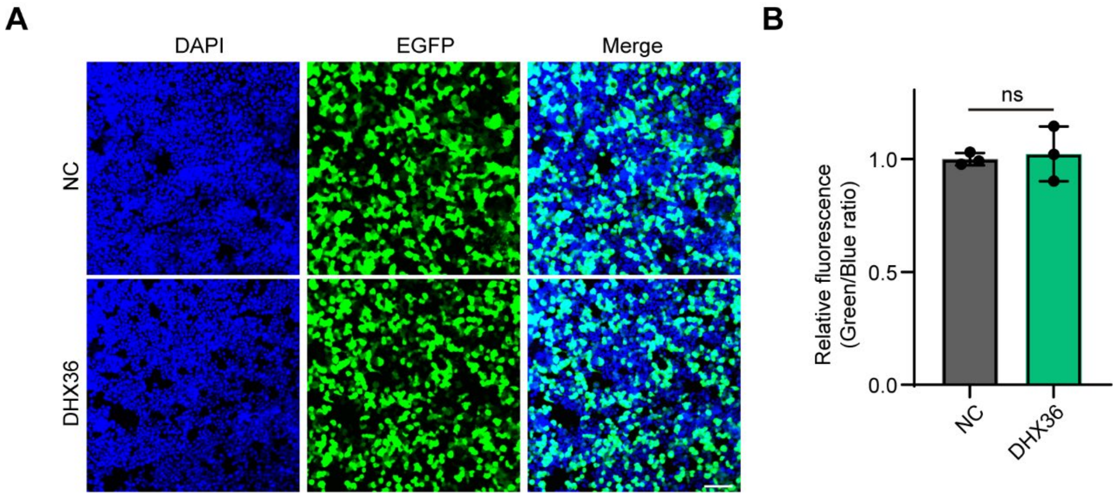

Figure S38. Effect of overexpressed DHX36 on MG-Mut-EGFP reporter protein. (A: Confocal pictures; B: Relative fluorescence intensity analyzed by image-J). All the experimental results were analyzed by three independent experiments, error bars represent S.E.M. SEM, standard error of mean significance was analyzed by t-test and scale bar was 100 μm. ns, no significant difference.

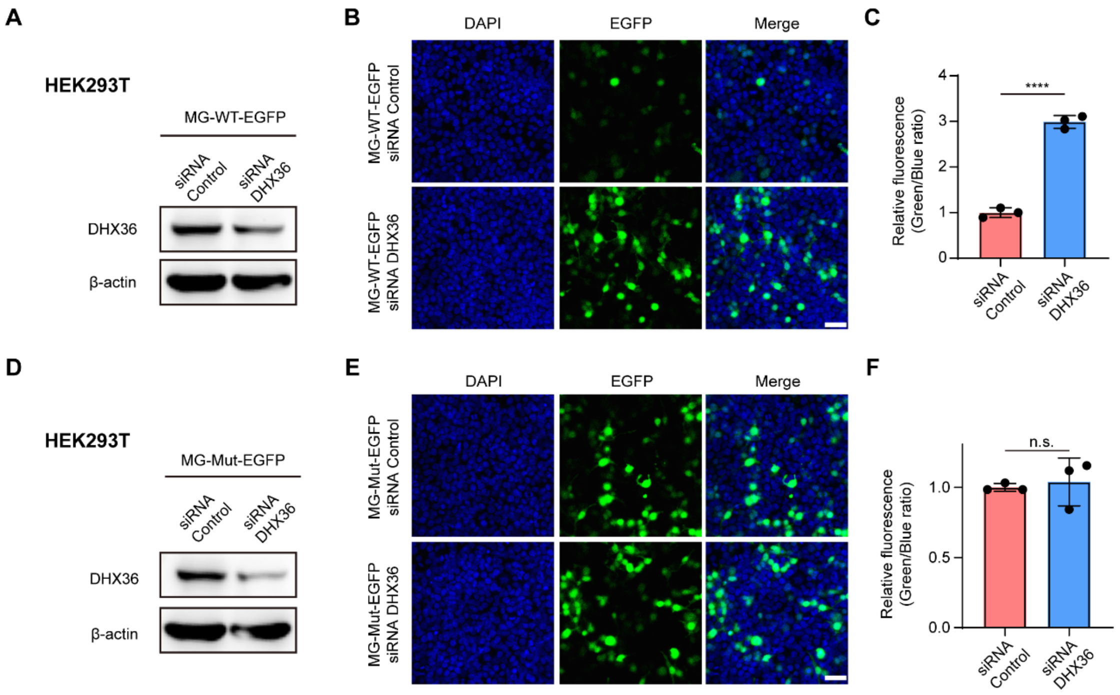

Figure S39. Effect of DHX36 knockdown on EGFP reporter. (A) The expression level of DHX36 protein in cells transfected with DHX36 siRNA and MG-WT-EGFP fluorescent reporter vector by western blot assay. (B-C) Effect of DHX36 knockdown on MG-WT-EGFP reporter protein. (B: Representative images of confocal pictures; C: Relative fluorescence intensity analyzed by image-J). (D) The expression level of DHX36 protein in cells transfected with DHX36 siRNA and MG-Mut-EGFP fluorescent reporter vector by western blot assay. (E-F) Effect of DHX36 knockdown on MG-Mut-EGFP reporter protein. (E: Representative images of confocal pictures; F: Relative fluorescence intensity analyzed by image-J). All the experimental results were analyzed by three independent experiments, error bars represent S.E.M. SEM, standard error of mean significance was analyzed by t-test and scale bar was 50 µm. ****, $p<0.0001$. ns, no significant difference.

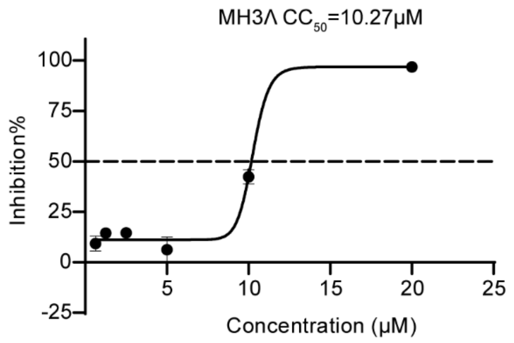

Figure S40. Inhibition activity curve of the compound against Vero cells.

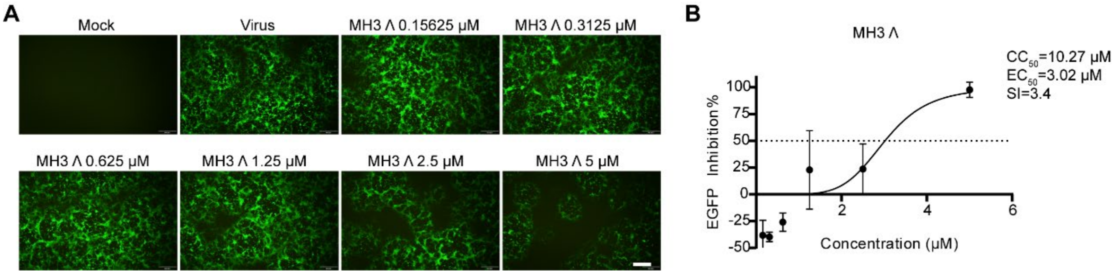

Figure S41. The effect of MH3 Λ on the fluorescence intensity of vaccinia virus TTV-EGFP. (A: Fluorescence images, Bars=500 mm; B: The fluorescence intensity inhibition rate curve calculated by image-J).

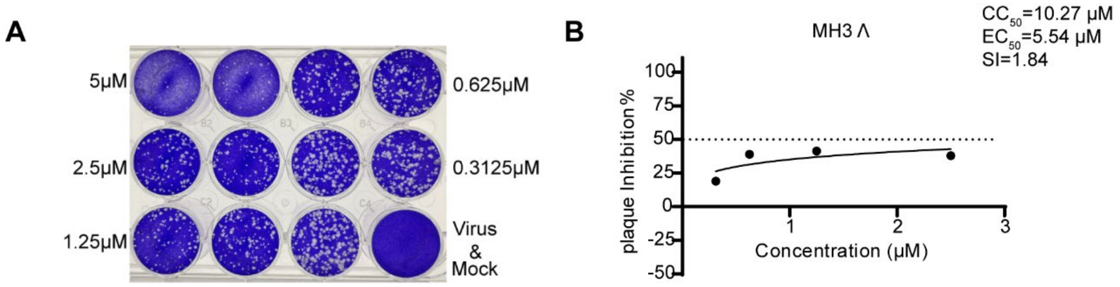

Figure S42. The effect of MH3Λ on vaccinia virus TTV-EGFP by plaque counting analysis. (A: plaque picture; B: plaque inhibition curve calculated by image-J.)

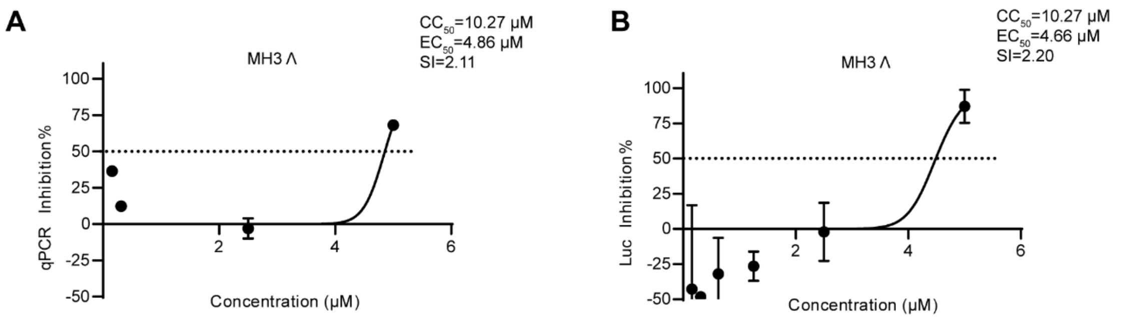

Figure S43. (A) The antiviral activity of MH3Λ against vaccinia virus TTV-EGFP by qPCR assay; (B) The antiviral activity of MH3Λ against vaccinia virus TTV-Luc was assessed by luciferase analysis.

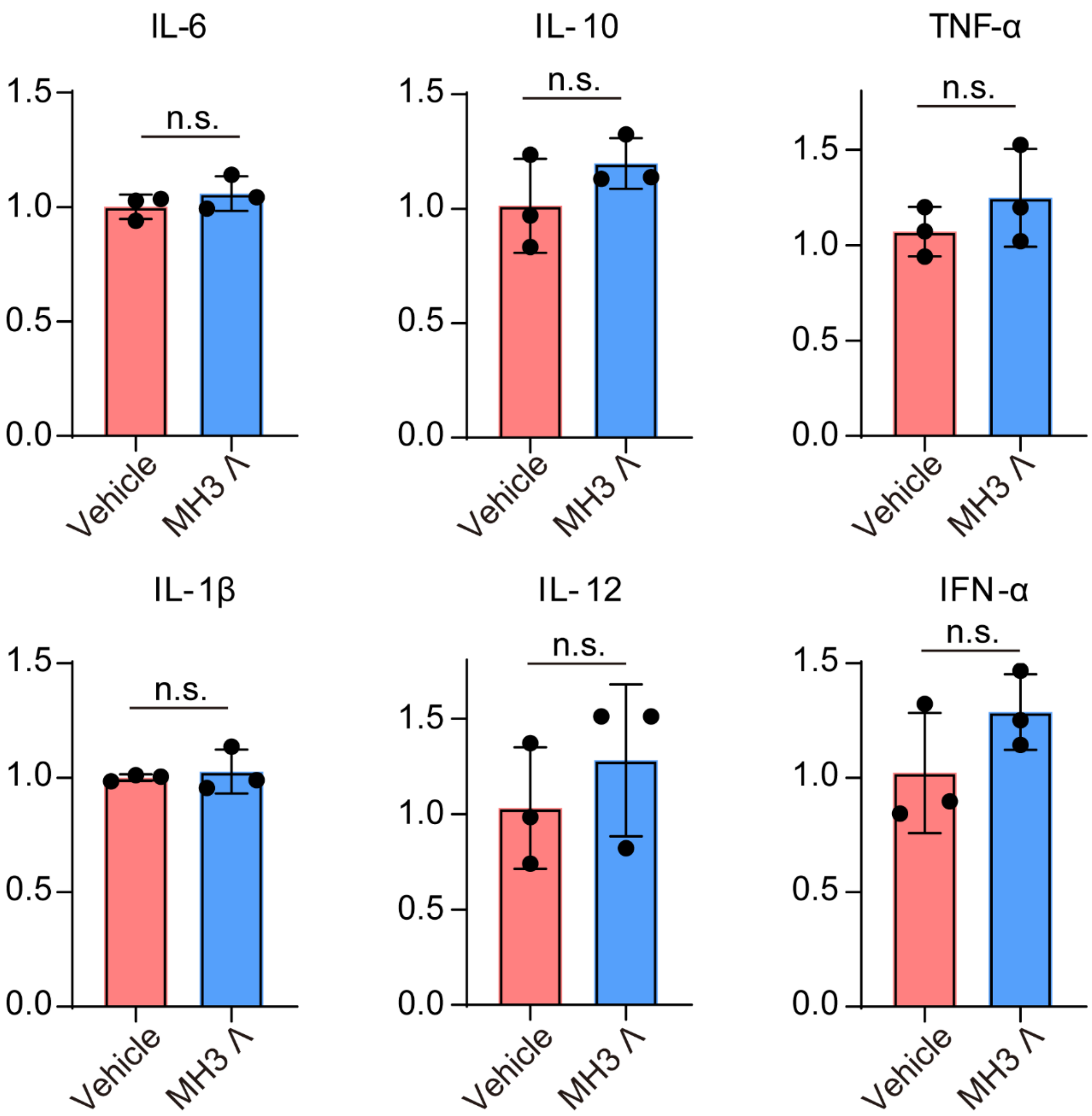

Figure S44 The effect of MH3 Λ on the mRNA expression levels of IL-6, IL-10, TNF-α, IL-1β, IL-12 and IFN-α in Vero cells via RT-qPCR. Data shown are mean±s.e.; n=3. n.s., not significant.

Table S1. Characteristics of the 29 putative RNA G-quadruplexes

Putative G4s	Position	Length	Sequence	G-Score	Gene	Protein
1	32139	20	GGAUUUGGUGCAAGGAUAGG	19	OPG048	Ribonucleotide reductase small subunit
2	33922	20	GGCGGUGUACGUGGACGAGG	15	OPG052	Cytoplasmic protein
3	46716	26	GGUGAUGCUGGUUCUGGAGAUUCUGG	18	OPG066	DNA-directed RNA polymerase 30 kDa polypeptide
4	61773	23	GGCUAUUCUGGCGGCUAGAAUGG	15	OPG080	Ribonucleoside-diphosphate reductase
5	65274	16	GGAGGAACCGGAGUGG	18	OPG084	RNA helicase NPH-II
6	70046	20	GGAUGGGUAAAAAAGGGAGG	16	OPG089	FEN1-like nuclease
7	73984	19	GGACCCGGUGGUCUUUCGG	16	OPG094	Myristylated protein
8	76733	20	GGUGGCUGAUUAGGCUGUGG	15	OPG097	Internal virion L3/FP4 protein
9	81240	25	GGUAGACUAGGUGCUAUGGAUGGGG	18	OPG105	DNA-dependent RNA polymerase subunit rpo147
10	83417	24	GGAUAUGGUGGUCGACGGAUACGG	18	OPG105	DNA-dependent RNA polymerase subunit rpo147
11	85906	18	GGAUGGUUAAAAAGGCGG	15	OPG107	Entry-fusion complex essential component
12	97300	23	GGUGUAUUGGACCUGGUAGACGG	19	OPG117	NTPase
13	111418	17	GGUUGGGUAGAUGGCGG	17	OPG130	39 kDa virion core protein
14	111634	20	GGAGCCGGUGUAGGUGUCGG	21	OPG130	39 kDa virion core protein
15	111682	20	GGUUGUAGCGGUUGGGUAGG	16	OPG130	39 kDa virion core protein
16	121262	23	GGACUUGGAUUCGGAUUUUGUGG	18	OPG139	IMV membrane protein A13L
17	124396	20	GGUAGAGGCAGUCGGAUUGG	19	OPG145	DNA helicase

18	130351	21	GGAAAGAGGUCCUCAGGUAGG	17	OPG151	DNA-dependent RNA polymerase subunit rpo132
19	130598	26	GGAUUCUUCGGUAACUUGGAGGUAGG	19	OPG151	DNA-dependent RNA polymerase subunit rpo132
20	136194	23	GGACAGUAGGUAAUGGCCAUGGG	19	OPG153	Orthopoxvirus A26L/A30L protein
21	136466	17	GGUGGUCUAGGAUGAGG	18	OPG153	Orthopoxvirus A26L/A30L protein
22	136487	20	GGUAUCGGAGUAGGUUUUGG	21	OPG153	Orthopoxvirus A26L/A30L protein
23	153961	28	GGUAAAGGAGGAAAGGGUGGUAUCAUGG	20	OPG180	DNA ligase
24	160688	17	GGAAAAGGUGGAUUCGG	18	OPG187	Ser/thr kinase
25	162516	16	GGAAUCAGGUGGGCGG	17	OPG188	Schlafen
26	171131	20	GGAGGAGAACAUGGAUAAGG	15	OPG199	Serpin
27	182709	23	GGUGCAAGUGGUGGUGUUGUAGG	15	OPG210	B22R family protein
28	183719	15	GGAGGUUGGUAAUGG	18	OPG210	B22R family protein
29	194858	23	GGAAUAGGAUACGGAGUAUCCGG	18	OPG002	Crm-B secreted TNF-alpha-receptor-like protein

2

Table S2. Name and sequence of the oligonucleotides and primers used in this study.

Oligomer	Sequence
MG	5'-GGUUGUAGCGGUUGGGUAGG-3'
MG-Mut	5'-GUUUGUAGCGUUUGUGUAGU-3'
F-MG	5'-FAM-GGUUGUAGCGGUUGGGUAGG-3'
F-MG -Mut	5' -FAM-GUUUGUAGCGUUUGUGUAGU-3'
F-MG-T	5'-FAM-GGUUGUAGCGGUUGGGUAGG-TAMRA-3'
F-MG-Mut-T	5' -FAM-GUUUGUAGCGUUUGUGUAGU-TAMRA-3'
F-Tel22-T	5'-FAM-AGGGTTAGGGTTAGGGTTAGGG-TAMRA-3'
F-BCL-2-T	5'-FAM-GGGCGCGGGAGGAATTGGGCGGG-TAMRA-3'
F-HIF1α-T	5'-FAM-AGGTGAGGCGGGCTTGCGGGA-TAMRA-3'
F-TBA-T	5'-FAM-GGTTGGTGTGGTTGG-TAMRA-3'
F-c-Myc-T	5'-FAM-TGAGGGTGGGTAGGGTGGGTAA-TAMRA-3'
F-c-Kit-1-T	5'-FAM-AGGGAGGGCGCTGGGAGGAGGG-TAMRA-3'
F-c-Kit-2-T	5'-FAM-CGGGCGGGCGCGAGGGAGGGG-TAMRA-3'
F-VEGF-A-T	5'-FAM-GGGGCGGGCCGGGGGCGGG-TAMRA-3'
F-RET-T	5'-FAM-AGGGGCGGGGCGGGGCGGGGG-TAMRA-3'
F-KRAS-T	5'-FAM-AGGGCGGTGTGGGAAGAGGGAAGAGGGGGAGG-TAMRA-3'
MPXV F3L-F	5'- CTCATTGATTTTTCGCGGGATA -3'
MPXV F3L-R	5'- CTCATTGATTTTTCGCGGGATA -3'
MPXV F3L-P	5′ FAM-CATCAGAATCTGTAGGCCGT-MGB-3'
TTV E3L -F	5'- ATCCTCTCTCATTGATTTTTCGCGGGA -3'
TTV E3L -R	5'- TGGAGAAGCGAGAAGTTAATAAAGC -3'
TTV E3L -P	5'- HEX- TCGTCGGAGCTGTACACCATAGCAC - BHQ1-3'
GDPDH-F	5'- AGAGTCAGCCGCGTCTTCTC -3'
GDPDH-R	5'- GTTAAAAGCAGCCCTGGTGA -3'
IL-1β-F	5'- CCATAGCCACATTTGGTT -3'
IL-1β-R	5'- AGGGACGCAGTTGCTCATC -3'
IL-6-F	5'- GTCTCCACAAGCGCCTTCGGTC -3'
IL-6-R	5'- GCTGTGTGGGGCGGCTACATCT -3'
IL-12-F	5'- CGGTCATCTGCCGCAAA -3'
IL-12-R	5'- TGCCCATTCGCTCCAAGA -3'
IL-10-F	5'- ATGCCCCAAGCTGAGAACCACGACCCA -3'
IL-10-R	5'- TCCCAAGGGGCTGGGTCAGGTATCCCA -3'
TNF-α-F	5'- GCCGCATCGCCGTCTCCTAC -3'
TNF-α-R	5'- CCTCAGCCCCCTCTGGAGTC -3'

MG-Mut-2	5’-GGUUGUAGCGAUUGGGUAGG -3’
IFN-α-F	5’- TTTCTTCTGCCTGAAGGACAG -3’
IFN -α-R	5’- TCTCATAATTTCTGCTCTGACA -3’

Table S3. The effect of addition of ligands or metallo-supramolecular complexes on DNA G4 stability.

Samples	ΔTm (℃) (DNA+MH3 Λ)	ΔTm (℃) (DNA+MH3 Δ)
BCL-2	0.35	0.34
Tel22	0.84	0.34
KRAS	0.30	-0.64
c-Kit-2	0.05	0.01
RET	-1.59	-0.99
c-Kit-1	1.31	0.42
VEGF-A	-0.08	0.20
TBA	0.10	0.02
c-Myc	-0.09	0.19
HIF1α	0.23	0.11

Table S4. The sequence of A5L-EGFP mRNA and the plasmid enhancer-promoter.

	**Sequence**
A5L-EGFP mRNA	ATGTTACTTTTGGAATCGTTCAAAACCTTTGACTAGTTGTATAATTTGATCTAT<br>TGCCCTACGCGTATACTCCCTTGCATCATATACGTTCGTCACCAGATCGTTTGT<br>TTCGGCCTGAAGTTGACGCATATCTTTTTCAACACTCGACATGAGATCCTTAA<br>GGGTCATATCGTCTAGATTTTGTTGAGATGCTGCTCCTGGATTTGGATTTTGTT<br>GTGCTGTTGTACATACTGTACCACCAGTAGGTGTGGGAGTACATACAGTGGCC<br>ACAATAGGAGGTTGAAGAGGTGTAACCGTTGGAGTAGTACAAGAAATACTTC<br>CATCCGATTGTTGTGTACATGTGGTTGTTGGTAACGTCTGAGAAGGTTGGGTA<br>GATGGCGGTGTCGTCATCTTTTGATCTTTATTAAATTTAGAGATAATATCCTGA<br>ACAGTATTGCTCGGCGTCAACGCTGGAAGGAGTGTACTCGCCGGCGCATCAGT<br>ATCTGCAGACAGCCAATCAAAAAGATTAGACATATCAGATGATGTATTAGTTT<br>GTTGTCGTGGTTTTAGTACAGGAGCAGTACTACTAGGTAGAAGAATAGGAGC<br>CGGTGTAGGTGTCGGAACCGGCTGTGGAGTTATATGAATAGTTGGTTGTAGCG<br>GTTGGGTAGGCTGTCTGCTGGCGACCATCATATTATCTCTAGCTAGTTGTTCTC<br>GCAACTGTCTTTGATAATACGACTCTTGAGACTTTAGTCCTATTTCAATCGCTT<br>CATCCTTTTTCGTATCCGGATCCTTTTCTTCAGAATAATAGATTGACGACTTTG<br>GTGTAGAGGATTCTGCCAGCCCCTGTGAGAACTTGTTAAAGAAGTCCATGTGA<br>GCAAGGGCGAGGAGCTGTTCACCGGGGTGGTGCCCATCCTGGTCGAGCTGGA<br>CGGCGACGTAAACGGCCACAAGTTCAGCGTGTCCGGCGAGGGCGAGGGCGAT<br>GCCACCTACGGCAAGCTGACCCTGAAGTTCATCTGCACCACCGGCAAGCTGCC<br>CGTGCCCTGGCCCACCCTCGTGACCACCCTGACCTACGGCGTGCAGTGCTTCA<br>GCCGCTACCCCGACCACATGAAGCAGCACGACTTCTTCAAGTCCGCCATGCCC<br>GAAGGCTACGTCCAGGAGCGCACCATCTTCTTCAAGGACGACGGCAACTACA<br>AGACCCGCGCCGAGGTGAAGTTCGAGGGCGACACCCTGGTGAACCGCATCGA<br>GCTGAAGGGCATCGACTTCAAGGAGGACGGCAACATCCTGGGGCACAAGCTG<br>GAGTACAACTACAACAGCCACAACGTCTATATCATGGCCGACAAGCAGAAGA<br>ACGGCATCAAGGTGAACTTCAAGATCCGCCACAACATCGAGGACGGCAGCGT<br>GCAGCTCGCCGACCACTACCAGCAGAACACCCCCATCGGCGACGGCCCCGTG<br>CTGCTGCCCGACAACCACTACCTGAGCACCCAGTCCGCCCTGAGCAAAGACC<br>CCAACGAGAAGCGCGATCACATGGTCCTGCTGGAGTTCGTGACCGCCGCCGG<br>GATCACTCTCGGCATGGACGAGCTGTACAAG
enhancer-promoter	CGTTACATAACTTACGGTAAATGGCCCGCCTGGCTGACCGCCCAACGACCCCC<br>GCCCATTGACGTCAATAATGACGTATGTTCCCATAGTAACGCCAATAGGGACT<br>TTCCATTGACGTCAATGGGTGGAGTATTTACGGTAAACTGCCCACTTGGCAGT<br>ACATCAAGTGTATCATATGCCAAGTACGCCCCCTATTGACGTCAATGACGGTA<br>AATGGCCCGCCTGGCATTATGCCCAGTACATGACCTTATGGGACTTTCCTACT

TGGCAGTACATCTACGTATTAGTCATCGCTATTACCATGGTGATGCGGTTTTG
GCAGTACATCAATGGGCGTGGATAGCGGTTTGACTCACGGGGATTTCCAAGTC
TCCACCCCATTGACGTCAATGGGAGTTTGTTTTGGCACCAAAATCAACGGGAC
TTTCCAAAATGTCGTAACAACTCCGCCCCATTGACGCAAATGGGCGGTAGGCG
TGTACGGTGGGAGGTCTATATAAGCAGAGCT

SI References

1. H. Song et al., Discovery of selective, antimetastatic and anti-cancer stem cell metallohelices via post-assembly modification. *Chem Sci* **10**, 8547-8557 (2019).
2. D. H. Simpson et al., Metallohelices that kill Gram-negative pathogens using intracellular antimicrobial peptide pathways. *Chem Sci* **10**, 9708-9720 (2019).
3. H. Song et al., Glycoconjugated Metallohelices have Improved Nuclear Delivery and Suppress Tumour Growth In Vivo. *Angew Chem Int Ed Engl* **59**, 14677-14685 (2020).
4. Huang B, Zhao H, Song J, Zhao L, Deng Y, Wang W, Lu R, Wang W, Ren J, Ye F, Tian H, Wu G, Ling H, Tan W. Isolation and Characterization of Monkeypox Virus from the First Case of Monkeypox - Chongqing Municipality, China, 2022. China CDC Wkly. 2022 Nov 18;4(46):1019-1024.
5. Wei Q, Huang B, Huang W, Song J, Mei L, Zhao L, Yin J, Zhang J, Wang W, Ye F, Li C, Bai L, Wang Y, Deng Y, Liu B, Jiang M, Wu G, Han J, Zhao H, Ling H, Tan W, Liu J. The first strain of monkeypox isolated in the Chinese Mainland and preserved at the National Pathogen Resource Center of China. *Infect Med (Beijing)*. 2022 Nov 19;1(4):288-291. doi: 10.1016/j.imj.2022.11.003.
